# Spatiotemporal regulation of insulin signaling by liquid–liquid phase separation

**DOI:** 10.1038/s41421-022-00430-1

**Published:** 2022-07-05

**Authors:** Kun Zhou, Qiaoli Chen, Jiamou Chen, Derong Liang, Weikuan Feng, Minjun Liu, Qi Wang, Ruizhen Wang, Qian Ouyang, Chao Quan, Shuai Chen

**Affiliations:** 1 MOE Key Laboratory of Model Animal for Disease Study and State Key Laboratory of Pharmaceutical Biotechnology, Department of Cardiology, Nanjing Drum Tower Hospital, Model Animal Research Center, School of Medicine, Nanjing University, Nanjing, Jiangsu China; 2 Jiangsu Key Laboratory of Molecular Medicine, Model Animal Research Center, School of Medicine, Nanjing University, Nanjing, Jiangsu China

**Keywords:** Insulin signalling, Mechanisms of disease

## Abstract

Insulin signals through its receptor to recruit insulin receptor substrates (IRS) and phosphatidylinositol 3-kinase (PI3K) to the plasma membrane for production of phosphatidylinositol-3,4,5-trisphosphate (PIP3) from phosphatidylinositol-4,5-bisphosphate [PI(4,5)P2], which consequently activates protein kinase B (PKB). How insulin signals transduce from the plasma membrane into the cytoplasm is not clearly understood. Here we show that liquid–liquid phase separation (LLPS) plays a critical role in spatiotemporal control of insulin signaling through regulating multiple components including IRS1. Both protein concentration and insulin stimulation can drive the formation of intracellular IRS1 condensates through LLPS. Components including PI(4,5)P2, p85-PI3K and PDK1 are constitutively present in IRS1 condensates whereas production of PIP3 and recruitment of PKB in them are induced by insulin. Thus, IRS1 condensates function as intracellular signal hubs to mediate insulin signaling, whose formation is impaired in insulin resistant cells. Collectively, these data reveal an important function of LLPS in spatiotemporal control of insulin signaling.

## Introduction

Insulin is a critical metabolic hormone, regulating many cellular processes to maintain cell functions and consequent metabolic health^1^. Insulin resistance, a condition in which cells display impaired insulin action, underlies the development of type 2 diabetes (T2D) that has become prevalent world-wide in the last few decades^2^. The prevalence of T2D urges a thorough understanding of mechanisms mediating insulin action and resistance.

Upon binding to insulin, the tyrosine kinase of insulin receptor (IR) undergoes activation through autophosphorylation on multiple tyrosine residues, and in turn phosphorylates the insulin receptor substrates (IRS)^3^. Phosphorylated IRS recruits the p85 regulatory subunit of phosphatidylinositol 3-kinase (p85-PI3K), and the p110 catalytic subunit consequently converts phosphatidylinositol-4,5-bisphosphate (PI(4,5)P2) into phosphatidylinositol-3,4,5-trisphosphate (PIP3)^4^. The signaling lipid PIP3 recruits downstream kinase effectors including the 3-phosphoinositide-dependent protein kinase-1 (PDK1) and protein kinase B (PKB, also known as Akt) to the plasma membrane, which allows PDK1 to be in close proximity with PKB to phosphorylate and activate PKB^5^. After activation by insulin, PKB in turn phosphorylates multiple substrates in various organs to deal with the postprandial surge of nutrients and ions. For instance, phosphorylation of GSK3 and PPP1R3G by PKB promotes glycogen deposition in the liver^6,7^, and PKB-mediated FoxO1 phosphorylation inhibits hepatic gluconeogenesis in response to insulin^8,9^. Insulin also stimulates uptake of glucose and fatty acids into skeletal muscle through phosphorylation of AS160 and RalGAPα1 by PKB^10–12^, and maintains calcium homeostasis in cardiac muscle via PKB-mediated phosphorylation of SPEG^13^. The diverse PKB substrates exhibit distinct subcellular localization in the cytoplasm whereas PKB activation occurs at the plasma membrane in this classical model. It is not entirely clear how active PKB efficiently reaches its substrates in the cytoplasm upon insulin stimulation.

Signaling proteins are often clustered into microdomains, e.g., PIP3-recruited PDK1 and PKB, to facilitate spatiotemporal control of signal transduction. Liquid–liquid phase separation (LLPS) allows proteins to form liquid-like membrane-less condensates, which acts as a molecular mechanism regulating diverse cellular processes^14^. Proteins in phase separated liquid droplets are mobile and undergo continuous exchange with the surrounding solutions^15^. LLPS proteins often contain intrinsically disordered regions (IDRs) with low complexities, and their phase separation is driven by multivalent, and often weak, interactions of IDRs^16^. Phase separation is concentration-dependent and regulated by posttranslational modifications such as protein phosphorylation^17^. The assembly of membrane-less condensates through LLPS can facilitate formation of signaling clusters and play important roles in signal transduction such as T cell receptor signaling and Wnt signaling^18,19^. However, very little is known about the relationship between LLPS and insulin signal transduction.

In this study, we investigate the potential relationship between LLPS and insulin signaling, and reveal that LLPS plays a critical role in spatiotemporal control of insulin signal transduction through regulating multiple signaling components. We show that IRS1 condensates resulted from LLPS may function as intracellular signal hubs to transduce insulin signals deep into the cells.

## Results

### Proteins with LLPS potential are enriched in the insulin signaling pathway

**Fig. 1:**
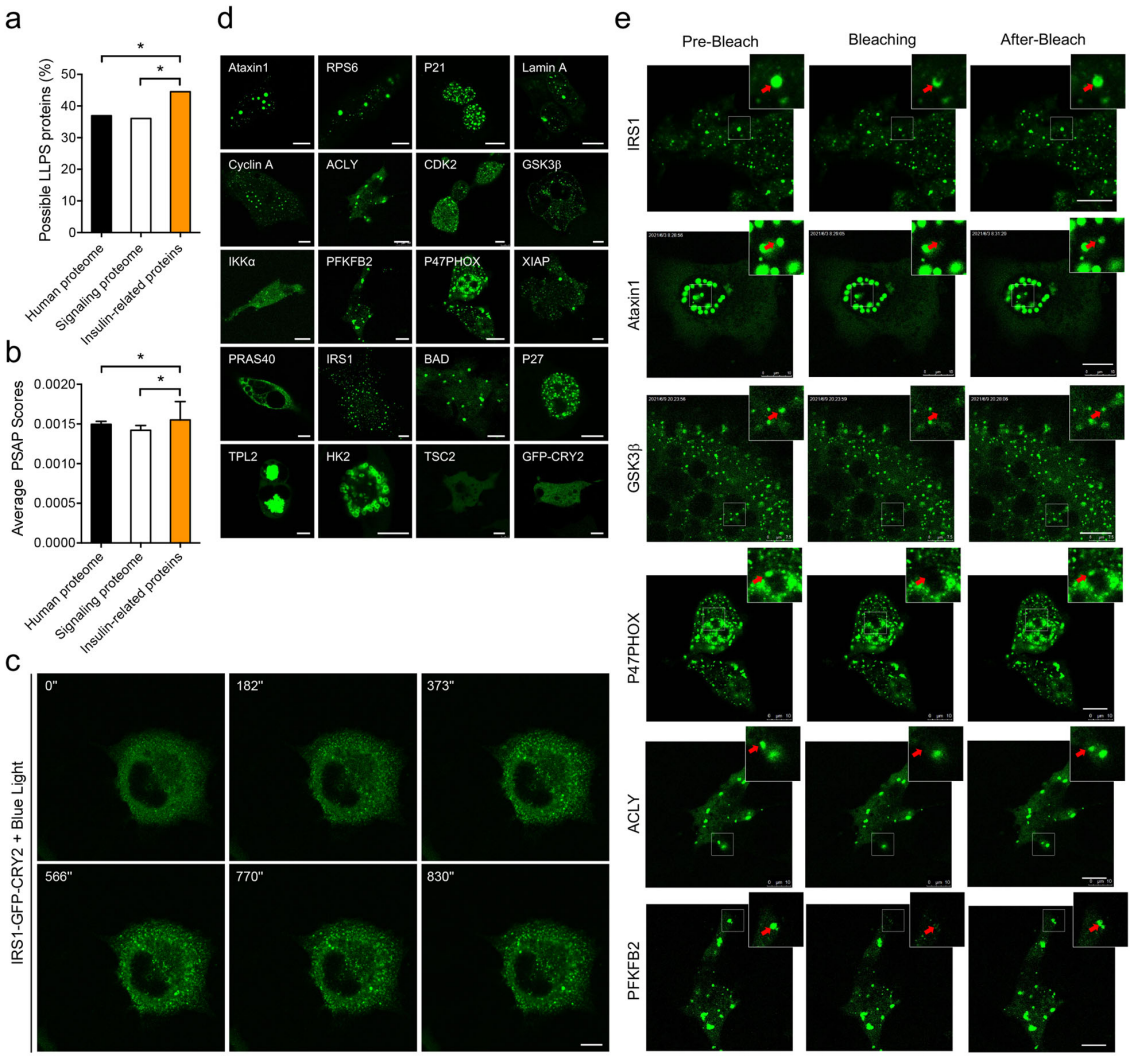
LLPS potential of insulin-related proteins. **a**, **b** LLPS scores of human proteome, signaling transduction proteome and insulin-related proteins. The predictor PSAP was used to calculate LLPS scores. The percentage of proteins with LLPS feature (**a**) and average store (**b**) were presented in the 3 groups. *n* = 17,817 (human proteome), 5520 (signaling transduction proteome), and 173 (insulin-related proteins). The data are given as percentage (**a**) and the means ± SEM (**b**). Statistical analyses were carried out using Fisher’s Exact test (**a**) and Mann–Whitney *U* test (**b**). *p < 0.05. **c** Time-series imaging of IRS1-GFP-CRY2 (optoIRS1) upon induction with blue light in Cos-7 cells. Scale bar, 10 μm. **d** Blue light induced formation of optoDroplets of insulin-related proteins in Cos-7 cells. Scale bar, 10 μm. **e** FRAP analysis of optoDroplets of insulin-related proteins in Cos-7 cells. The optoDroplets subjected to FRAP analysis were highlighted in insets. Scale bar, 10 μm.

We first sought to find out which components of the insulin signaling pathway might have potential to undergo LLPS. KEGG pathway analysis revealed 173 insulin-related proteins including the receptor, mediators and effectors of insulin signaling pathway (Supplementary Table S1), which were subjected to bioinformatics analysis using an LLPS predictor PASP^20^. 77 out of 173 insulin-related proteins were predicted to possess LLPS potential (Supplementary Table S1). The percentage (44.5%) of proteins with LLPS potential in the insulin signaling pathway was significantly higher than the one (36.9%) in human proteome (Fig. 1a, b). When the insulin signaling pathway was compared to signal transduction in general, the percentage of proteins with LLPS potential was still higher in the insulin signaling pathway (Fig. 1a, b). These data suggest that proteins with LLPS potential are enriched in the insulin signaling pathway.

We next investigated the phase separation capacity of insulin signaling proteins identified via PASP using a mammalian cell system. To this end, we employed an optoDroplet system, in which target proteins were fused to green fluorescent protein (GFP) and the Cry2PHR domain, to enable blue light activation of phase separation of target proteins in living cells^21^. Fusing IRS1 to Cry2PHR (optoIRS1) resulted in rapid blue light-dependent phase separation in cells (Fig. 1c). Prior to illumination with blue light, optoIRS1 protein was evenly distributed in cells. Notably, bright foci of optoIRS1 started to emerge within minutes upon illumination with blue light and continued to enlarge over time, showing that optoIRS1 displayed blue light-dependent condensation. Similarly, blue light-mediated cluster assemblies were also observed with optoP47PHOX, optoACLY, optoIKKα and optoCyclinA, in cells (Supplementary Video S1a–d). In total, we examined 19 insulin signaling components using the optoDroplet system, and found that all of them except the optoTSC2 exhibited blue light-mediated condensation (Fig. 1d). One feature of liquid droplets formed by protein phase separation is the fluidity within the droplets, which facilitates fluorescence recovery when an inner region of the droplets is photo-bleached using a laser. We then studied the fluidity of these condensates using the fluorescence recovery after photobleaching (FRAP) technique, and found that the fluorescent signals were recovered after the inner regions of optoDroplets were photo-bleached for 15 of the above optoProteins including optoIRS1, optoATXN1 and optoGSK3β, suggesting that these proteins undergo LLPS under this experimental condition. In contrast, two optoProteins, namely optoHK2 and optoTPL2, formed gel-like condensates or immobile aggregates, and their fluorescent signals could not recover in the FRAP experiment (Fig. 1e; Supplementary Fig. S1a–c). Together, these data suggest that LLPS may play a role in mediating insulin signal transduction.

### Validation of LLPS proteins of insulin signaling in vitro

**Fig. 2:**
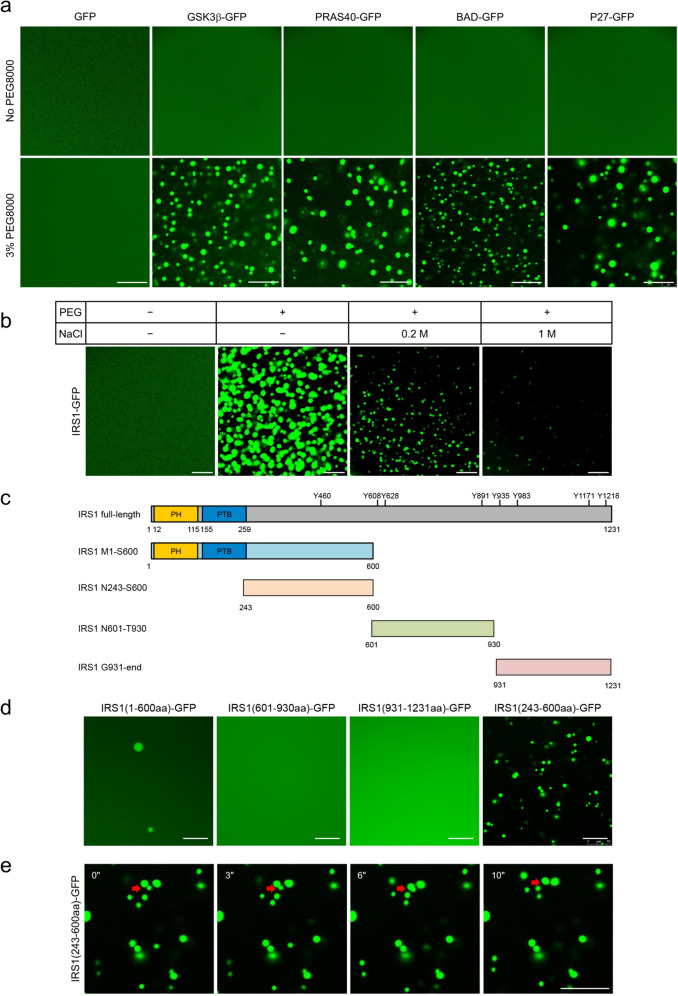
Validation of LLPS proteins of insulin signaling in vitro. **a** In vitro LLPS assay with 20 μM recombinant His-tagged proteins GFP, GSK3β-GFP, PRAS40-GFP, BAD-GFP, and P27-GFP with or without 3% PEG8000. Scale bar, 10 μm. **b** In vitro LLPS assay with recombinant His-tagged full-length IRS1-GFP with 3% PEG8000 ± NaCl. Scale bar, 10 μm. **c** Schematic illustration of domains and fragments of mouse IRS1. Eight tyrosine sites regulated by insulin receptor are shown in the diagram. **d** In vitro LLPS assay with recombinant His-tagged four fragments IRS1-GFP with 3% PEG8000. Scale bar, 10 μm. **e** Fusion of droplets of His-tagged IRS1^N243-S600^-GFP. Red arrows showed the two droplets that fused during the experimental period. Time Zero started at 90 s after the addition of PEG8000. Scale bar, 10 μm.

Since the light-sensitive Cry2PHR can self-associate upon the exposure to blue light, the optoDroplet system might artificially modulate intracellular protein interactions to cause LLPS. Therefore, we employed an in vitro LLPS assay to further study the LLPS potential of these insulin signaling components. To this end, we fused a His-GFP dual tag to 5 of the above proteins, namely IRS1, GSK3β, PRAS40, BAD and p27, and expressed them in *E. coli*. The purified recombinant proteins were used for an in vitro LLPS assay, in which phase separation was initiated by adding PEG8000. As a negative control, the His-GFP fusion protein did not form droplets before and after the addition of PEG8000 (Fig. 2a). None of the target proteins displayed phase separation prior to the addition of PEG8000 (Fig. 2a, b). Notably, the addition of PEG8000 induced the formation of droplets for His-GFP-tagged IRS1, GSK3β, PRAS40, BAD, and p27 in solution (Fig. 2a, b). PEG8000-induced condensation of these recombinant proteins occurred very fast normally within seconds. These protein droplets were quite mobile presumably due to Brownian motion, and smaller protein droplets could fuse with each other to form larger droplets (Supplementary Fig. S2a). The rapid fusion of these protein droplets is a good indicator of their fluidity. To further study the biophysical property of IRS1 condensates, we examined its droplet formation under varying salt concentrations, and found that PEG8000-induced phase transition of His-GFP-tagged full-length IRS1 was weakened with the increased concentrations of NaCl (Fig. 2b). To rule out the possibility that GFP might contribute to the PEG8000-induced LLPS when fused to the target proteins, we expressed and purified His-tagged GSK3β, PRAS40, BAD and p27 in *E. coli* and Flag-tagged IRS1 in HEK293 cells for the in vitro LLPS assay after labeled with FITC. Again, the addition of PEG8000-induced condensation of the FITC-labeled His-GSK3β, His-PRAS40, His-BAD and His-p27 (Supplementary Fig. S2b). Under this in vitro condition, FITC-labeled Flag-IRS1 started to form condensates with a minimal concentration of 2 μM after the addition of PEG8000 (Supplementary Fig. S2c). Next, we fragmented IRS1 into three parts, an N-terminus IRS1^M1-S600^, a middle region IRS1^N601-T930^ and a C-terminus IRS1^G931-end^, and fused them with the His-GFP dual tag (Fig. 2c). Only the N-terminus IRS1^M1-S600^, but not the IRS1^N601-T930^ and IRS1^G931-end^, exhibited a relatively weak droplet-formation ability in vitro (Fig. 2d). This N-terminus IRS1^M1-S600^ contains a pleckstrin homology (PH) domain and a phospho-tyrosine binding (PTB) domain within the 1-259 aa region, and a IDR spanning from 243 aa to 600 aa (Fig. 2c). Notably, the His-GFP-tagged IRS1^N243-S600^ fragment displayed an enhanced ability to form droplets when PEG8000 was added into solutions as compared to the IRS1^M1-S600^ (Fig. 2d). The protein droplets of IRS1^N243-S600^ were also mobile and could grow bigger through fusion with each other (Fig. 2e). This IDR spanning from N243 to S600 on IRS1 is also found in the other three IRSs. Together, these data demonstrate that insulin-responsive IRS1, GSK3β, PRAS40, BAD, and p27 possess sequences with the intrinsic LLPS activity.

### Spontaneous formation of protein droplets of IRS1 in cells

**Fig. 3:**
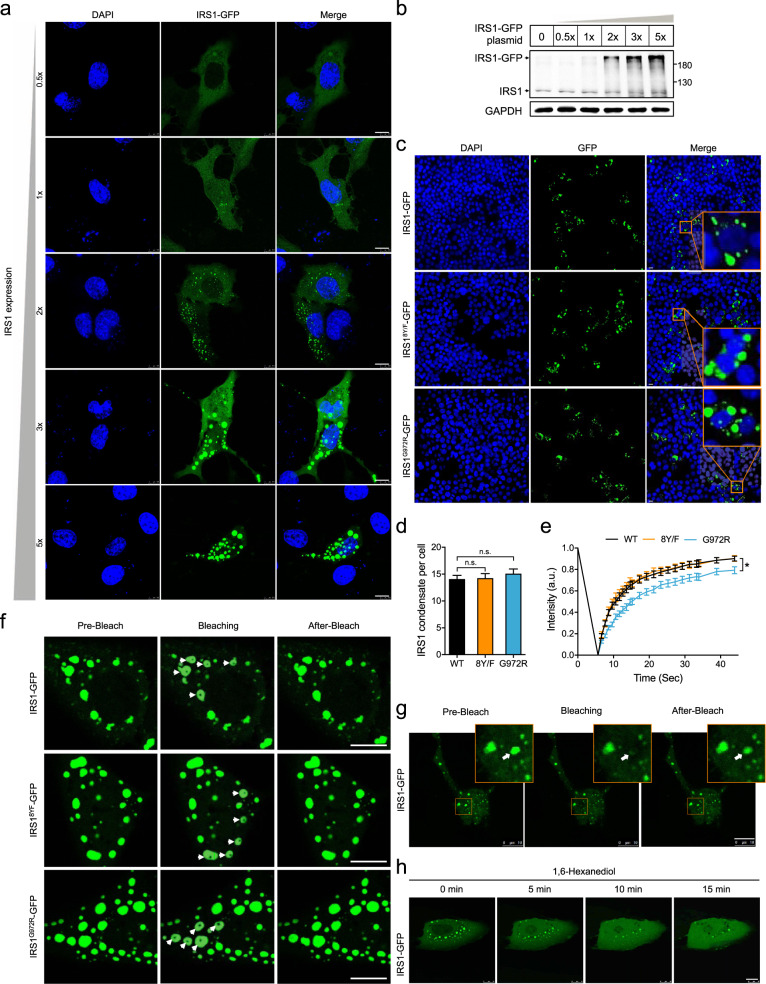
Concentration-dependent formation of IRS1-GFP condensates in cells. **a**, **b** Concentration-dependent formation of IRS1-GFP condensates in U2OS cells. Various amounts of IRS1-GFP plasmid were transfected into cells (1× IRS1-GFP indicates 0.4 μg plasmid DNA per well in a 12-well plate) to achieve increased expression levels of IRS1-GFP protein in U2OS cells. The formation of IRS1-GFP condensates was detected via fluorescence confocal microscope (**a**), and the expression levels of IRS1-GFP and endogenous IRS1 were determined via immunoblotting (**b**). Scale bar, 10 μm. **c**, **d** Formation of condensates of IRS1-GFP or IRS1^8Y/F^-GFP or IRS1^G972R^-GFP in HEK293T cells. The 8 tyrosine residues whose phosphorylation is dependent on IR were mutated to non-phosphorylatable phenylalanine residues on IRS1^8Y/F^-GFP. **c** Representative images. **d** Quantification of IRS1-GFP condensates per cell. *n* = 113–156. Statistical analyses were carried out via one-way ANOVA. n.s., not significant. Scale bar, 10 μm. **e**, **f** FRAP analysis of wild-type or mutant IRS1-GFP condensates in U2OS cells. **e** Quantification of FRAP analyses. **f** Representative images. Scale bar, 10 μm. The condensates subjected to FRAP were indicated with white arrows. The ROI intensity was normalized to the value before photo-bleaching and given as the means ± SEM. *n* = 34–43. Statistical analyses were carried out via one-way ANOVA. *p < 0.05 (IRS1^G972R^-GFP vs. WT IRS1-GFP, IRS1^G972R^-GFP vs IRS1^8Y/F^-GFP). **g** The material exchange between IRS1-GFP condensates and cytoplasm was examined by FRAP analysis in U2OS cells. The whole condensate of IRS1-GFP was photo-bleached and monitored subsequently. Scale bar, 10 μm. **h** Effects of 1,6-hexanediol on IRS1-GFP condensates. 1,6-hexanediol (5%) was added to U2OS cells, and IRS1-GFP condensates were monitored subsequently. Scale bar, 10 μm.

IRS1 is a central node in mediating insulin signaling, and we then focused on its regulation by LLPS. IRS1-GFP was expressed from low to high levels in U2OS cells through transfection with different amounts of IRS1-GFP encoding cDNA (Fig. 3a, b). When IRS1-GFP was expressed at the low level (with 1× plasmid DNA) that was comparable to endogenous IRS1 with an estimated concentration of ~50 nM, it was evenly diffused within cells with few small spherical foci formed in the cytosol (Fig. 3a, b). As IRS1-GFP was expressed to higher levels, its even distribution was decreased while its spherical foci were increased in both numbers and sizes in a concentration-dependent manner (Fig. 3a, b). This concentration-dependent foci formation did not require IR-dependent tyrosine phosphorylation of IRS1. A mutant IRS1 protein with the eight IR-dependent tyrosine residues mutated to phenylalanine (IRS1^8Y/F^) formed spherical foci in a concentration-dependent manner, similar as the wild-type IRS1 (Fig. 3c). Human G972R substitution (G965R on mouse IRS1) is the most common mutation of IRS1, which aggravates insulin resistance in obese patients^22^. The GFP-tagged IRS1^G972R^ mutant could also form spherical foci in cells, which were similar to the wild-type IRS1 and IRS1^8Y/F^ mutant (Fig. 3c). The foci numbers exhibited no difference between the wild-type IRS1 and two IRS1 mutants (Fig. 3d). The distribution pattern of IRS1 with characteristics of both dispersion and condensation was distinct from those of IR, p85-PI3K and PKB (Supplementary Fig. S3a). The IRS1 condensates were not associated with the plasma membrane (Supplementary Fig. S4a, b), and did not co-localize with intracellular organelles including endoplasmic reticulum, golgi apparatus, mitochondria, lysosome, early endosome and late endosome (Supplementary Fig. S4c). We reconstructed the IRS1-GFP foci in cells via 3D scanning, and the 3D images from different angles demonstrated that the IRS1-GFP foci were three-dimensional spheres rather than planar plates (Supplementary Fig. S3b). The IRS1-GFP spherical foci were present mainly in the cytosol, and could also be found in the nucleus but to a lesser extent when expressed at high levels (Fig. 3a; Supplementary Fig. S3c). Furthermore, the oblique cutting of the 3D-reconstructed IRS1-GFP condensates showed that they were farctate rather than hollow spheres (Supplementary Fig. S3b).

Our next question was whether the IRS1-GFP protein in the condensates was also mobile as the optoIRS1 in its droplets. FRAP analysis revealed that the fluorescence signals lost at the bleaching site was recovered within a minute, which demonstrates the fluidity within the IRS1-GFP condensates (Fig. 3e, f). Similarly, the fluorescence signals could be recovered even when the whole IRS1-GFP condensate was bleached, showing that active protein exchange occurs between the IRS1-GFP condensates and their surrounding cytoplasm (Fig. 3g). 1,6-Hexanediol is a solvent that is widely used to dissolve LLPS condensates but not protein gels and intracellular vesicles^23^. Notably, IRS1-GFP condensates in cells were gradually dissolved within 15 min after treatment with 1,6 hexanediol (Fig. 3h), again suggesting an LLPS-nature of these condensates. The IRS1^8Y/F^-GFP condensates exhibited the fluidity similar to the wild-type IRS1-GFP in the FRAP assay (Fig. 3e, f). In contrast, the recovery of fluorescence signals after photobleaching was slower in the IRS1^G972R^-GFP condensates than that in the wild-type or 8Y/F mutant IRS1 condensates (Fig. 3e, f), suggesting that the IRS1^G972R^ mutation impaired the fluidity of IRS1 condensates.

### Insulin regulates dynamics of protein droplets of IRS1

**Fig. 4:**
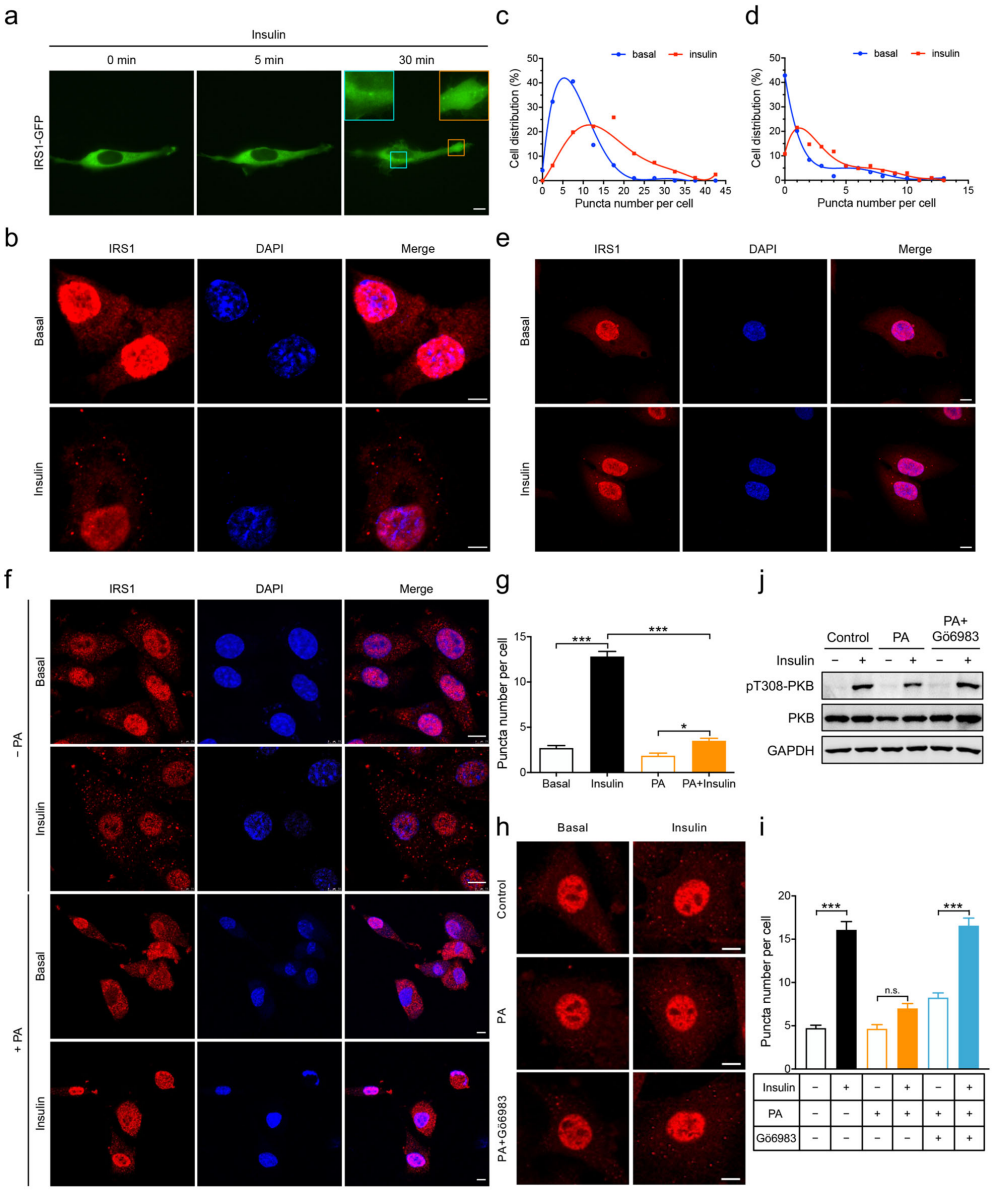
Insulin regulates dynamics of IRS1 condensates in cells. **a** Dynamics of IRS1-GFP condensates in U2OS cells upon insulin stimulation. Cells transfected with IRS1-GFP were stimulated with insulin (100 nM) for indicated time. Insets show the formation of IRS1-GFP condensates. Scale bar, 10 μm. **b**, **c** Formation of endogenous IRS1 condensates in L6 muscle cells upon insulin stimulation. **b** Representative images. **c** Fitted curves of cell distribution in terms of IRS1 condensate (puncta) number per cell. The values were fitted into curves using polynomials. *n* = 81–96. Scale bar, 5 μm. **d**, **e** Formation of endogenous IRS1 condensates in U2OS cells upon insulin stimulation. **d** Fitted curves of cell distribution in terms of IRS1 condensate (puncta) number per cell. The values were fitted into curves using polynomials. *n* = 102–119. **e** Representative images. Scale bar, 10 μm. **f**, **g** Effects of palmitate (PA) treatment on insulin-induced formation of IRS1 condensates in L6 muscle cells. Cells were treated with or without palmitate (400 μM) for 24 h before stimulated with or without insulin (100 nM). **f** Representative images. Scale bar, 10 μm. **g** Quantification of IRS1 puncta per cell. *n* = 50. The data are given as the means ± SEM. Statistical analyses were carried out via two-way ANOVA. *p < 0.05, and ***p < 0.001. **h**–**j** Effects of the PKC inhibitor Gö6983 on PKB phosphorylation and IRS1 puncta formation in L6 muscle cells. Cells were treated with or without palmitate for 24 h before stimulated with or without insulin in the presence or absence of Gö6983 (1 μM). **h** Representative images of IRS1 puncta. Scale bar, 10 μm. **i** Quantification of IRS1 puncta per cell. *n* = 55-62. **j** Immunoblotting analysis of PKB phosphorylation. The data are given as the means ± SEM. Statistical analyses were carried out via two-way ANOVA. ***p < 0.001. n.s., not significant.

We next sought to find out whether IRS1 protein condensates underwent dynamic changes upon insulin stimulation in cells. To this end, we first expressed IRS1-GFP in U2OS cells at a low level, which resulted in an even distribution of IRS1-GFP within cells (Fig. 4a). Insulin stimulation for 5 min did not cause apparent condensation of IRS1-GFP whereas 30 min treatment induced a number of bright green foci, suggesting formation of IRS1-GFP condensates (Fig. 4a). We then performed an immunofluorescence assay to examine endogenous IRS1 condensates in various cells including rat L6 muscle cells, U2OS cells, African green monkey Cos-7 kidney fibroblast-like cells and mouse primary brown adipocytes. Similar as previously reported^24^, immunofluorescent signals for endogenous IRS1 were found in both cytoplasm and nucleus of these cells (Fig. 4b–e; Supplementary Fig. S5a–d). Immunoblotting analyses revealed that three endogenous IRS1 isoforms were present in the cytosol with molecular weights of ~180, ~130, and ~115 kDa while a dominant IRS1 isoform existed in the nucleus with a size of ~115 kDa (Supplementary Fig. S3d, e). In contrast, the exogenous IRS1-GFP was mainly expressed in the cytosol with a molecular weight of ~200 kDa (Supplementary Fig. S3f). In L6 muscle cells, endogenous IRS1 displayed an even distribution in the cytosol with few foci under basal conditions (Fig. 4b, c). Importantly, insulin stimulation increased the number of cytosolic IRS1 foci in L6 muscle cells, suggesting an induction of endogenous IRS1 condensates (Fig. 4b, c). Less endogenous IRS1 condensates were found in U2OS cells under both basal and insulin stimulation conditions as compared to those in L6 muscle cells. Nevertheless, insulin stimulation also increased cytosolic puncta of endogenous IRS1 in U2OS cells (Fig. 4d, e). Similar effects of insulin on endogenous IRS1 condensates were observed in primary brown adipocytes and Cos-7 cells (Supplementary Fig. S5a–d). Together, these data demonstrate that insulin regulates dynamics of IRS1 protein condensates in cells.

### Palmitate inhibits insulin-induced formation of IRS1 condensates

Palmitate inhibits insulin signaling transduction through the IRS1-PI3K-PKB pathway thereby causing insulin resistance in various cell types^25^. As expected, treatment of L6 muscle cells with palmitate attenuated insulin-stimulated phosphorylation of PKB, showing that palmitate induced insulin resistance in these cells (Supplementary Fig. S5e). Again, insulin stimulation induced the formation of cytosolic puncta of endogenous IRS1 in L6 muscle cells that were not pre-treated with palmitate (Fig. 4f, g). Notably, pre-treatment of L6 muscle cells with palmitate greatly inhibited insulin-induced formation of endogenous IRS1 puncta in the cytosol (Fig. 4f, g), which was reversed by the addition of a PKC inhibitor Gö6983 (Fig. 4h, i). Concomitantly, the addition of Gö6983 restored insulin-stimulated PKB phosphorylation in palmitate-treated L6 muscle cells (Fig. 4j). A similar effect of palmitate on endogenous IRS1 condensates was observed in mouse primary brown adipocytes and U2OS cells. Palmitate pre-treatment blunted insulin-induced cytosolic condensation of endogenous IRS1 in brown adipocytes and U2OS cells (Supplementary Fig. S5f–i). The addition of Gö6983 restored insulin-induced formation of IRS1 condensates and PKB phosphorylation in palmitate-treated U2OS cells (Supplementary Fig. S5h–j). Together, these data show that palmitate inhibits insulin-induced formation of endogenous IRS1 condensates, which is in parallel with the development of insulin resistance in cells. PKC may be the key factor mediating the effect of palmitate on the formation of IRS1 condensates in cells.

### IRS1 condensates are functional hubs mediating insulin signaling

**Fig. 5:**
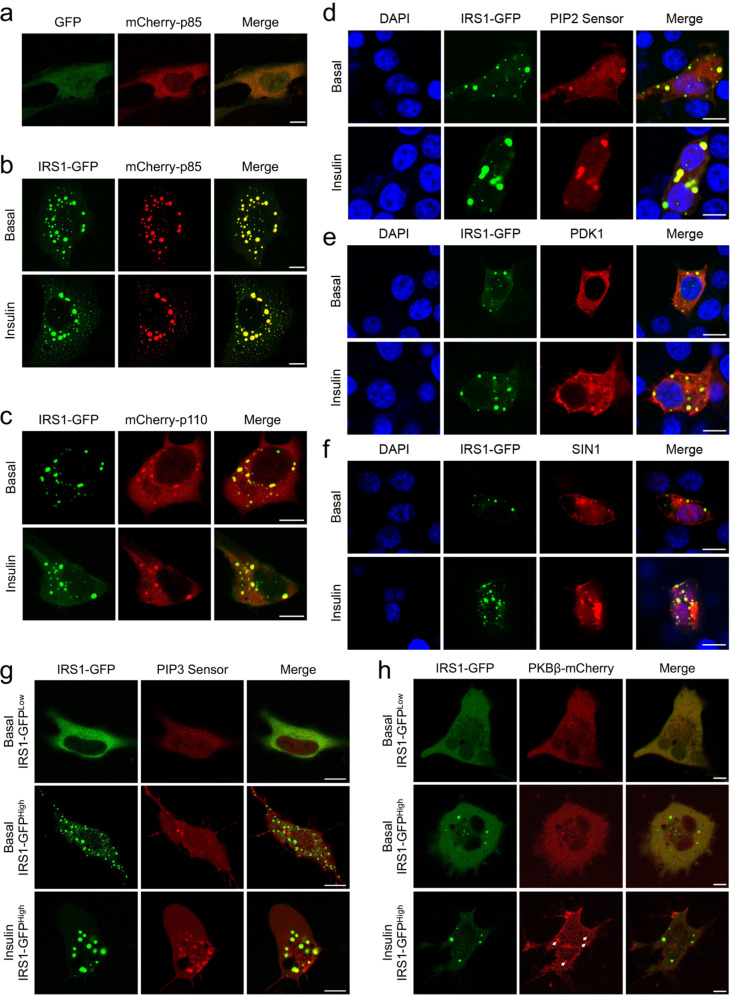
Recruitment of signaling molecules and PIP3 production in IRS1-GFP condensates. **a** Cellular localization of mCherry-p85 with co-expressed GFP in U2OS cells. Scale bar, 10 μm. **b**, **c** Cellular localization of IRS1-GFP co-expressed with mCherry-p85 (**b**) and mCherry-p110 (**c**) in response to insulin stimulation in U2OS cells. Scale bar, 10 μm. Quantification of colocalization was shown in Supplementary Fig. S6. **d**–**f** Cellular localization of IRS1-GFP co-expressed with PIP2 sensor (**d**), mCherry-PDK1 (**e**), and mCherry-SIN1 (**f**) in response to insulin stimulation in HEK293T cells. Scale bar, 10 μm. Quantification of colocalization was shown in Supplementary Fig. S6. **g** Cellular localization of IRS1-GFP co-expressed with PIP3 sensor in response to insulin stimulation. IRS1-GFP was expressed at low and high levels in U2OS cells. Scale bar, 10 μm. Quantification of colocalization was shown in Supplementary Fig. S6. **h** Cellular localization of IRS1-GFP co-expressed with PKBβ-mCherry in response to insulin stimulation. IRS1-GFP was expressed at low and high levels in Cos-7 cells. Arrows indicated PKBβ-mCherry that was co-localized with IRS1 condensates upon insulin stimulation. Scale bar, 10 μm. Quantification of colocalization was shown in Supplementary Fig. S6.

**Fig. 6:**
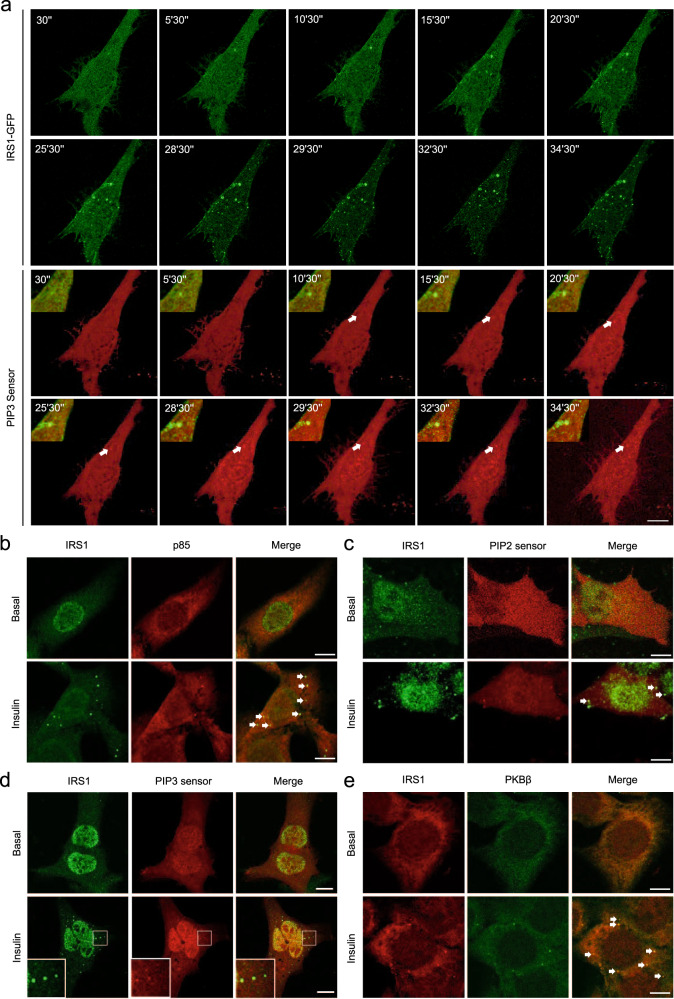
IRS1 condensates produced PIP3 and recruited PKB in response to insulin. **a** Time-series imaging of IRS1-GFP and PIP3 sensor in U2OS cells upon insulin stimulation. The merged images of IRS1-GFP and PIP3 sensor were enlarged and shown in the upper left corners. Scale bar, 10 μm. **b** Cellular localization of endogenous IRS1 and p85 in U2OS cells in response to insulin stimulation. The IRS1 puncta co-localized with p85 were indicated with white arrows. The p85 antibody was labeled with Alexa564. Scale bar, 10 μm. **c** Cellular localization of endogenous IRS1 and PIP2 sensor in U2OS cells in response to insulin stimulation. The IRS1 puncta co-localized with PIP2 sensor were indicated with white arrows. Scale bar, 10 μm. **d** Cellular localization of endogenous IRS1 and PIP3 sensor in U2OS cells in response to insulin stimulation. The IRS1 puncta co-localized with PIP3 sensor were indicated with white arrows. Scale bar, 10 μm. **e** Cellular localization of endogenous IRS1 and PKBβ in U2OS cells in response to insulin stimulation. The IRS1 puncta co-localized with PKBβ were indicated with white arrows. The IRS1 antibody was labeled with Alexa564. Scale bar, 10 μm.

Protein condensates resulted from LLPS can recruit related factors for enrichment to promote the efficiency of enzymatic reactions^26,27^. Therefore, we hypothesized that IRS1 condensates might recruit other insulin signal components to mediate signal transduction. It is well established that IRS1 interacts with the p85 regulatory subunit of PI3K through its phosphorylated tyrosine residues upon insulin stimulation. The p85-mCherry exhibited a diffused expression pattern when co-expressed with GFP in cells (Fig. 5a). However, when co-expressed with a high level of IRS1-GFP that spontaneously-formed condensates, p85-mCherry was found almost exclusively in these IRS1-GFP condensates (Fig. 5b). Notably, p85-mCherry was present in these spontaneously-formed IRS1-GFP condensates regardless of insulin stimulation (Fig. 5b; Supplementary Fig. S6a). Similarly, the mCherry-tagged p110 catalytic subunit of PI3K was constitutively present in the IRS1-GFP condensates irrespective of insulin treatment (Fig. 5c; Supplementary Fig. S6b). PI3K utilizes phosphatidylinositol 4,5,-bisphosphate [PI(4,5)P2] to yield phosphatidylinositol 3,4,5,-trisphosphate (PIP3)^4^. Interestingly, a PI(4,5)P2 sensor revealed the presence of PI(4,5)P2 in the spontaneous IRS1-GFP condensates in the absence of insulin stimulation (Fig. 5d; Supplementary Fig. S6c). We then constructed a PIP3 sensor that diffused within the cells at the basal state but was enriched at the plasma membrane upon insulin stimulation (Supplementary Fig. S7). The PIP3 sensor also formed intracellular foci in response to insulin stimulation, suggesting the existence of PIP3 generation centers inside the cells. In contrast to PI(4,5)P2, PIP3 was not present in the spontaneous IRS1-GFP condensates in the absence of insulin stimulation (Fig. 5g; Supplementary Fig. S6f). Notably, insulin treatment resulted in marked production of PIP3 not only at the plasma membrane but also within these spontaneous IRS1-GFP condensates (Fig. 5g; Supplementary Fig. S6f). Moreover, a single-cell tracking experiment revealed that insulin-induced newborn IRS1-GFP condensates could also produce PIP3 (Fig. 6a).

PDK1 and mTORC2 are two critical kinases activating PKB in response to insulin stimulation. Similar as PI3K and PI(4,5)P2, mCherry-tagged PDK1 and an mTORC2 subunit SIN1 were present the spontaneous IRS1-GFP condensates even in the absence of insulin stimulation (Fig. 5e, f; Supplementary Fig. S6d, e). Expression of mCherry-PKBβ was diffused in cells co-expressing a low level of IRS1-GFP that did not form condensates (Fig. 5h). In cells co-expressing a high level of IRS1-GFP that formed condensates, mCherry-PKBβ remained as a diffused protein and was not recruited into the spontaneous IRS1-GFP condensates in the absence of insulin stimulation (Fig. 5h; Supplementary Fig. S6g). Notably, mCherry-PKBβ expectedly became associated with the plasma membrane, and was recruited into the spontaneous IRS1-GFP condensates upon insulin stimulation (Fig. 5h; Supplementary Fig. S6g).

Moreover, insulin induced the formation of endogenous IRS1 condensates, which simultaneously recruited endogenous p85 and PIP2 into the condensates and promoted the production of PIP3 and the appearance of endogenous PKB in the IRS1 granules (Fig. 6b–e).

Taken together, these data show that IRS1 condensates may function as signal hubs scattered inside the cells to mediate insulin signaling.

### The PH-PTB domains recruit PIP2 into IRS1 condensates for PIP3 generation

**Fig. 7:**
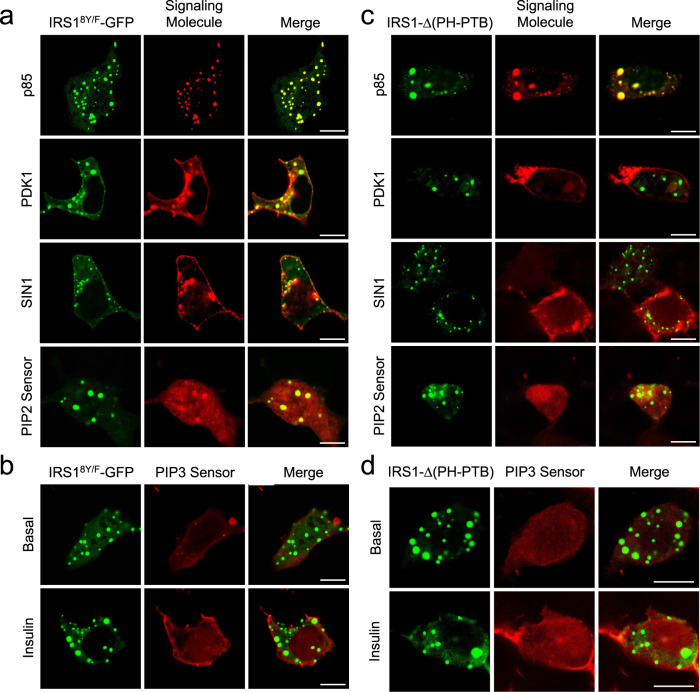
Recruitment of signaling molecules and PIP3 production in mutant IRS1 condensates. **a** Cellular localization of IRS1^8Y/F^-GFP co-expressed with mCherry-p85, mCherry-PDK1, mCherry-SIN1 and PIP2 sensor in HEK293T cells. Scale bar, 10 μm. **b** Cellular localization of IRS1^8Y/F^-GFP co-expressed with PIP3 sensor in response to insulin stimulation in HEK293T cells. Scale bar, 10 μm. **c** Cellular localization of IRS1Δ(PH-PTB)-GFP co-expressed with mCherry-p85, mCherry-PDK1, mCherry-SIN1, and PIP2 sensor in HEK293T cells. Scale bar, 10 μm. **d** Cellular localization of IRS1Δ(PH-PTB)-GFP co-expressed with PIP3 sensor in response to insulin stimulation in HEK293T cells. Scale bar, 10 μm.

We next examined how the insulin signaling components were assembled into the spontaneous IRS1-GFP condensates. In agreement with the constitutive presence of p85-mCherry in the spontaneous IRS1-GFP condensates (Fig. 5b; Supplementary Fig. S6a), p85-mCherry was still present in the condensates of the mutant IRS1^8Y/F^-GFP (Fig. 7a), further showing that the recruitment of p85-mCherry did not depend on the tyrosine phosphorylation of IRS1. Similarly, PIP2, PDK1 and SIN1 were all present in the IRS1^8Y/F^-GFP condensates (Fig. 7a). The p85 regulatory subunit exerts an inhibitory effect on the p110 catalytic subunit, and the binding of p85 to phosphopeptides relieves its inhibition towards p110^28^. Notably, insulin could no longer stimulate the production of PIP3 in the IRS1^8Y/F^-GFP condensates (Fig. 7b), which is in contrast to that in the wild-type IRS1-GFP condensates (Fig. 5g). These data suggest that the tyrosine phosphorylation of IRS1 is not required for recruitment of p85 into the IRS1 condensates but indispensable for activation of p85/p110 dimers. Notably, the G972R mutation significantly decreased the recruitment of p85 into the IRS1 condensates (Supplementary Fig. S8a, b), which was concomitant with the impaired fluidity of the condensates (Fig. 3e, f).

The PH and PTB domains of IRS1 can bind PIP2^29^, which might be a source of PIP2 in the IRS1 condensates. To test this hypothesis, we generated an IRS1-Δ(PH-PTB) mutant in which the PH-PTB domains were deleted. This IRS1-Δ(PH-PTB) mutant could still spontaneously form condensates in cells, which could recruit p85 but failed to recruit PIP2 (Fig. 7c). Concomitantly, the IRS1-Δ(PH-PTB) condensates could no longer recruit the PIP2-binding PDK1 and SIN1 (Fig. 7c). Moreover, insulin stimulation did not induce PIP3 production in the IRS1-Δ(PH-PTB) condensates (Fig. 7d). These data show that IRS1 condensates recruits PIP2 through the PH-PTB domains, which may in turn recruit PDK1 and SIN1 and is the major source of substrates for PI3K to generate PIP3.

## Discussion

**Fig. 8:**
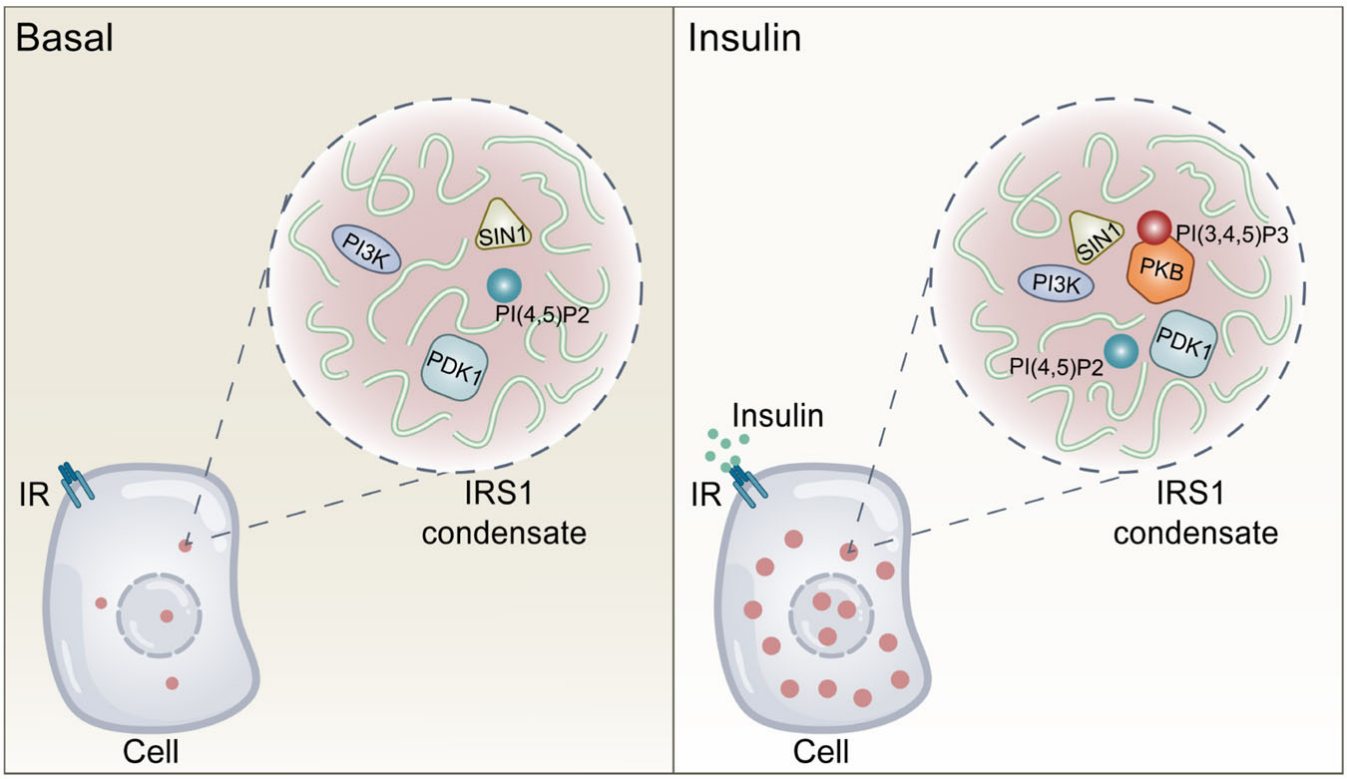
A model of IRS1 condensates as intracellular insulin signal hubs. A schematic illustration of IRS1 condensates serving as intracellular signal hubs to execute insulin signal transduction. IRS1 condensates form through LLPS in concentration- or insulin-dependent manners. At the basal state, multiple components including PI(4,5)P2, PI3K, PDK1, and SIN1 are present in IRS1 condensates. Upon insulin stimulation, more components such as PIP3 and PKB appear in IRS1 condensates to mediate insulin signal transduction.

Our findings demonstrate that LLPS is an important feature enriched in insulin-related components and plays a critical role in mediating insulin signaling. Our data is consistent with a model in which IRS1 condensates resulted from LLPS may act as signal hubs scattered inside the cells to execute insulin signal transduction (Fig. 8).

Spatiotemporal control of formation of signal clusters is critical to ensure high efficiency and specificity for signal transduction. It has been well established that insulin signals through its receptor to recruit IRS1-PI3K-PDK1-PKB to the plasma membrane to form signal clusters, thereby exerting its physiological effects^4^. Once activated through phosphorylation by PDK1 and mTORC2 at the plasma membrane, PKB can then dissociate from there to move into the cytosol and nucleus to phosphorylate its downstream targets^30^. It has long been recognized that IRS1 is present in some low-density fractions in the cytosol, which is distinct from IR- or GLUT4-residing vesicles^31,32^. In light of our findings, IRS1 in the previously-reported low-density fractions might be its condensates formed through LLPS in the cytosol. Therefore, our findings on IRS1 condensates as insulin signal hubs provide an alternative mechanism showing that insulin-related signal clusters or signalosomes can be formed in the cytosol, and possibly in the nucleus as well, thus explaining how insulin efficiently signals deep into the cells.

The LLPS-mediated formation of IRS1 condensates can be initiated through at least two driving forces, IRS1 expression levels and insulin stimulation. The concentration-dependent LLPS of IRS1 does not require tyrosine phosphorylation of IRS1. Instead, the IDR spanning from N243 to S600 is sufficient to drive LLPS in vitro. Though the C-terminal region itself does not undergo LLPS, it might modulate the condensation through the N-terminal IDR. The concentration-dependent LLPS of IRS1 might be an important source of pre-existing IRS1 condensates in cells in the absence of insulin, and might be of significance under certain physiological or pathological conditions. For example, IRS1 expression is up-regulated in certain tumors^33,34^, which raises a possibility that IRS1 condensates might be associated with cancer development and progression. Insulin is another driving force for LLPS of IRS1 through mechanisms that still need to be defined. One possibility is that IR is internalized after activation by insulin^35^ and recruits IRS1 to increase its local concentration thereby leading to formation of IRS1 condensates through LLPS. In such a scenario, IRS1 condensates would have a vesicular core with IR on it. However, our data show that IRS1 condensates are farctate rather than hollow spheres. Furthermore, it has been reported that IRS1-containing low-density fractions in the cytosol are distinct from IR-residing vesicles^31^. Nevertheless, further investigation is required to clarify this possibility. Another possibility is that insulin signals through its components at the plasma membrane to induce LLPS of IRS1 possibly via posttranslational modification (PTM). PTMs such as phosphorylation, ubiquitination and sumoylation are important in regulation of LLPS^14^. For example, mTORC1-mediated phosphorylation of PGL-1/-3 promotes LLPS of PGL granules in response to heat stress^17^.

Another critical question regarding the signal hub of IRS1 condensates is their components that mediate insulin signal transduction. Certain insulin signaling components are constitutively present in the pre-existing IRS1 condensates, including PI(4,5)P2, PI3K, PDK1, and SIN1. IRS1 contains the PH-PTB domains that can bind PI(4,5)P2^29^, which may bring this phosphoiositide species into IRS1 condensates during LLPS. Alternatively, the IRS1 condensates might make transient contact with certain membranes to obtain PI(4,5)P2 through the PH-PTB domains of IRS1 after condensate formation. PDK1 and mTORC2 are two key kinases phosphorylating Thr^308^ and Ser^473^ on PKB, respectively, thereby activating the latter kinase^36,37^. PDK1 also possesses a PH domain that exhibits strong interaction with PIP3 or PI(4,5)P2, which results in its constitutive localization with the plasma membrane^38^. It is possible that PDK1 is constitutively recruited into the IRS1 condensates via PI(4,5)P2 that is enriched in these condensates. Alternatively, it might be recruited into IRS1 condensates through direct or indirect protein-protein interaction with condensed IRS1. Similarly, SIN1, a key component of mTORC2, is also a PH domain-containing protein that can bind to PIP3 or PI(4,5)P2^39^, whose recruitment into IRS1 condensates also remains to be defined. Despite the constitutive presence of p85-PI3K and PI(4,5)P2 in IRS1 condensates, the generation of PIP3 in them is still dependent on insulin stimulation. The insulin-dependent production of PIP3 may play several roles in signal transduction in IRS1 condensates. PIP3 can recruit PKB into IRS1 condensates through the PH domain on PKB. Similar to the production of PIP3, recruitment of PKB into IRS1 condensates is also insulin-dependent. PIP3 binding to the PH domain of PKB also elicits conformational changes to expose its activation loop to be phosphorylated by PDK1^40^. Furthermore, the binding of PDK1 to PIP3 facilitates the rate at which PDK1 phosphorylates and activates PKB^38^. Lastly, the binding of PIP3 to the PH domain of SIN1 releases its inhibition of mTORC2 activity towards phosphorylating PKB^39^.

IRS1 belongs to a small protein family that has three other members, IRS2-4^41^. IRS1 and IRS2 are the major IRS expressed in all tissues while IRS3 and IRS4 exhibit a restricted tissue distribution^41,42^. The IDR on IRS1 (N243-S600) mediating the LLPS in vitro is also present in the other three IRSs. It would be intriguing to find out whether IRS2-4 can form condensates through LLPS to function as signal hubs. These IRSs function as adaptor proteins in signal transduction downstream of receptor tyrosine kinases such as IR, insulin-like growth factor-1 receptor (IGF1R) and interleukin-4 receptor (IL-4R)^43^. Our findings therefore put forward new questions whether all these receptor tyrosine kinases regulate the formation of IRS condensates through LLPS, and whether IRS condensates function as intracellular signal hubs mediating signal transduction downstream of these receptor tyrosine kinases.

IRS1 plays critical roles not only in the transduction of insulin signals but also in the development of insulin resistance. The classical view on these roles of IRS1 focuses on its PTMs in which tyrosine phosphorylation of IRS1 by IR mediates insulin signaling while serine phosphorylation of IRS1 by PKC, JNK, IKKβ or S6K antagonizes its tyrosine phosphorylation thereby resulting in insulin resistance^2^. Our data reveal a previously-unrecognized mechanism for IRS1 to mediate insulin signaling and to confer insulin resistance, which involves LLPS-mediated formation of IRS1 condensates. These IRS1 condensates function as intracellular signal hubs to transduce insulin signals deep into the cells. The IRS1^G972R^ mutation that causes human insulin resistance does not affect the formation of IRS1 condensates but decreases their fluidity in concomitance with impaired recruitment of p85 into the condensates. Palmitate treatment, a known factor causing insulin resistance, impairs the formation of the signal hubs of IRS1 condensates, which might contribute to the development of insulin resistance. The palmitate-impaired IRS1 condensate formation is mediated by PKC, and can be restored by the treatment of the PKC inhibitor Gö6983. Therefore, our findings may help to develop new strategies for drug discovery to combat metabolic diseases associated with insulin resistance. The discovery of IRS1 condensates as signal hubs for the PI3K-PKB pathway may also have impacts on other diseases such as cancer.

In summary, we show that LLPS plays a critical role in the transduction of insulin signals through regulating multiple signaling components. Our data reveal that LLPS-derived IRS1 condensates may function as intracellular signal hubs to execute insulin signal transduction and that impaired formation of IRS1 condensates is associated with insulin resistance.

## Materials and methods

### Materials

Recombinant human insulin was purchased from Novo Nordisk (Bagsvaerd, Denmark). 1,6-Hexanediol was from Sangon Biotech (Cat No. A100159), and PEG8000 from Sangon Biotech (Cat No. A601513). Live-cell nucleus dye Hoechst from Beyotime Biotech (Cat No. C1022). Ni-NTA resin (Cat No. SA004010) was bought from Smart-Lifesciences (Changzhou, China). Palmitate was from Sigma-Aldrich (Cat No. P9417). All other chemicals were purchased from Sigma-Aldrich (Shanghai, China) or Sangon Biotech (Shanghai, China).

### Antibodies

The rabbit antibody against IRS1 (Cat No. 17509-1-AP) and GAPDH (Cat No. 60004-1-Ig) were bought from Proteintech (Wuhan, China). The rabbit antibody against p85 (ab191606) was from Abcam, and the rabbit antibody for PKBβ (HPA064521) was from Atlas Antibodies. The antibodies that recognize pS473-PKB (Cat No. 9271), pT308-PKB (Cat No. 13038), total PKB (Cat No. 9272), LAMP1 (Cat No. 9091), PDI (Cat No. 3501), RCAS1 (Cat No. 12290) were from Cell Signaling Technology. The antibodies that recognize total TOM20 (Cat No. sc-514625), Rab5 (Cat No. sc-46692), Rab7 (Cat No. sc-376372) was from Santa Cruz (Dallas, Texas, USA). The Lamin A/C antibody (Cat No. A0249) was from Abclonal (Wuhan, China).

### Molecular biology

The cDNAs encoding mouse IRS1 (NP_034700.2), human GSK3β (NP_002084.2), human RPS6 (NP_001001.2), human P27(NP_004055.1), human BAD (NP_004313.1), human PRAS40 (NP_115751.3), human CDK2 (NP_001789.2), human Ataxin1 (NP_000323.2), human XIAP (NP_001158.2), human TSC2 (NP_000539.2), human Cyclin A (NP_001228.2), human HK2 (NP_000180.2), human PFKFB2 (NP_006203.2), human Lamin A (NP_733821.1), human IKKalpha (NP_001269.3), human 14-3-3 (NP_006817.1), human ACLY (NP_001087.2), human TPL2 (NP_005195.2), human P47PHOX (NP_000256.4), mouse SIN1 (NP_001277554.1),mouse PDK1 (NP_035192.2), mouse P85alpha (NP_001070963.1), human PKB betta (NP_001617.1) were cloned into pcDNA5-FRT/TO vectors with tags, for expression in mammalian cells. The cDNAs encoding mouse IRS1 (NP_034700.2), human GSK3β (NP_002084.2), human P27 (NP_004055.1), human BAD (NP_004313.1), human PRAS40 (NP_115751.3), human CDK2 (NP_001789.2) were cloned into pET vectors with tags, for expression in *E. coli*. Standard cloning procedures were carried out to introduce point mutations or to make fragmentation. All plasmids were sequenced at AZENTA Life Science (Suzhou, China).

### Recombinant protein expression and purification

The recombinant proteins with 6× His tag were expressed in *E. coli* (BL21DE3), and purified using Ni-NTA beads. Briefly, after transfection with plasmids, *E. coli* were cultured to a density with OD600 between 0.6 and 0.8, and then induced with IPTG (Sangon Biotech) for protein expression at 18 °C overnight. Afterwards, bacteria were collected through centrifugation and ultrasonically lysed in the lysis buffer (50 mM Tris-Cl (pH = 7.4), 10 U/mL DNaseI, 5 mM DTT, 0.2 mM PMSF, 1 μg/mL leupeptin, 1 mM benzamidine, 1 mM aprotinin, 1 mM pepstatin). The resultant lysates were incubated with Ni-NTA beads overnight. After washing in a wash buffer (50 mM Tris-Cl (pH = 7.4), 5 mM DTT, 0.2 mM PMSF, 1 μg/mL leupeptin, 1 mM benzamidine, 1 mM aprotinin, 1 mM pepstatin, 20 mM imidazole, 150 mM NaCl) for 3 times, recombinant proteins were eluted from beads and stored in a buffer (50 mM Tris-Cl (pH = 7.4), 5 mM DTT, 0.2 mM PMSF, 1 μg/mL leupeptin, 1 mM benzamidine, 1 mM aprotinin, 1 mM pepstatin, 200 mM imidazole, 150 mM NaCl, 10% glycerol).

Flag-IRS1 expressed in HEK293 cells was captured using the Flag beads, and then eluted with the Flag peptide.

### In vitro LLPS assay

Purified recombinant proteins with the GFP tag were concentrated using Amicon Ultra Centrifugal tubes (Sigma-Aldrich), and used for the in vitro LLPS assay. Recombinant proteins (9 μL) were dropped onto glass dishes (NEST), and imaged with a fluorescence confocal microscopy. Formation of condensates in the solution was initiated by the addition of 1 μL PEG8000 (30%). His-tagged recombinant proteins or Flag-IRS1 were labeled with FITC and used in the in vitro LLPS assay.

### Construction of phosphatidylinositide sensors

The PH domain of mouse PLCδ1 (NP_001280577) was cloned into pcDNA5-FRT/TO vectors with a mCherry tag, and used as a PIP2 sensor. The PH domain of human PKBβ (NP_001617.1) was cloned to into pcDNA5-FRT/TO vectors with the mCherry tag, and used as a PIP3 sensor.

### Mouse breeding and husbandry

The Ethics Committee at Model Animal Research Center of Nanjing University approved the animal protocols used in this study. Mice were produced and maintained in a specific pathogen free animal facility.

### Isolation of primary brown adipocytes

Primary brown preadipocytes were isolated from 1-month-old wild-type mice. BAT was cut into small pieces and digested in a digestion buffer (1× HBSS, 2% BSA and 0.2% type II collagenase) for 20 min at 37 °C. The primary preadipocytes were then passed through cell strainers (40 µm mesh size) and span down by centrifugation for 5 min at 150× *g* and re-suspended with culture medium. Once cells reached confluence, preadipocytes were cultured in differentiation medium (DMEM supplemented with 10% fetal bovine serum (FBS), 850 nM insulin, 0.5 μM Dexamethasone, 250 μM IBMX, 1 μM Rosiglitazone, 2 nM T3, 650 μM Indomethacin) for 2 days, and further differentiated in DMEM containing 10% FBS, 160 nM insulin and 2 nM T3 for 2 days. Then adipocytes were cultured in maintenance medium (DMEM supplemented with 10% FBS, 2 nM T3) that were changed every 2 days. After differentiation, adipocytes were treated with or without PA.

### Cell culture, transfection, and stimulation

Human embryonic kidney HEK293 cells, U2OS cells and African green monkey fibroblast Cos-7 cells were obtained from the Cell Resource Center, Chinese Academy of Medical Sciences and Peking Union Medical College (China). Rat L6 myoblasts were from Dr. Amira Klip (University of Toronto, Canada). Cells were cultured in DMEM or McCoy’s 5A medium containing 10% (v/v) foetal bovine serum and subjected to mycoplasma contamination test on a regular basis. Cells were transfected with plasmid DNA using Lipofectamine 3000 reagent (Thermo Fisher Scientific), and further cultured for 1 day before imaging. L6 myoblasts were treated with or without palmitate (400 μM) for 24 h, and then stimulated with or without insulin (100 nM) for 30 min before lysis or imaging.

### Cell lysis

L6 myoblasts were harvested and lysed in a lysis buffer containing 50 mM Tris-HCl (pH 7.4), 1 mM EDTA, 1 mM EGTA, 1% (v/v) Triton X-100, 1 mM sodium ortho-vanadate, 10 mM sodium glycerophosphate, 50 mM sodium fluoride 5 mM sodium pyrophosphate, 0.27 M sucrose, 2 µM microcystin-LR, 1 mM benzamidine, 0.1% (v/v) 2-mercaptoethanol, 0.2 mM phenylmethanesulfonyl fluoride, 1 mg/mL Leupeptin, 1 mg/mL Pepstatin and 1 mg/ml Aprotinin. Protein concentrations of cell lysates were measured using Bradford reagent (Thermo Fisher Scientific).

### Immunoblotting

Immunoblotting was carried out as previously described^13^. Briefly, proteins were immunoblotted onto nitrocellulose membranes after eletrophoretically separated via SDS-PAGE. Membranes were blocked with 5% milk, and sequentially incubated with primary antibodies and horseradish-peroxidase-conjugated secondary antibodies. Unbound secondary antibodies were removed from membranes through intensive washes. ECL substrates (GE Healthcare, UK) were then added, and chemiluminescence signals were recorded using a gel documentation system (Tanon, China).

### Imaging

After transfection, cells cultured on coverslips were fixed with 4% paraformaldehyde, and used for imaging of fluorescent proteins using a Leica SP5 fluorescence confocal microscope or GE DeltaVision elite fluorescence microscope. Epi- and TIRF-images were taken using a GE DeltaVision OMX microscope with a TIRF module. For immunofluorescence imaging of endogenous IRS1, cells were fixed and blocked in a blocking buffer (goat serum from Boster Biological Technology) for 1 h, and further incubated with IRS1 antibody (Proteintech, Cat No. 17509-1-AP) overnight at 4 °C. On the following day, cells were washed, incubated with fluorophore-conjugated secondary antibodies for 1 h at room temperature in the dark together with DAPI. After intensive washes, the coverslips with cells were mounted onto slides, and imaging were carried out using the Leica SP5 fluorescence confocal microscope or GE DeltaVision elite fluorescence microscope. For co-staining of endogenous IRS1/p85, fixed cells were first stained with the IRS1 primary antibody and fluorophore-conjugated secondary antibody, and then incubated with the Alexa564-labeled p85 antibody (Abcam, ab191606). For co-staining of endogenous IRS1/PKBβ, fixed cells were first incubated with the PKBβ primary antibody (Atlas Antibodies, HPA064521) and fluorophore-conjugated secondary antibody, and then stained with the Alexa564-labeled IRS1 antibody.

For live-cell imaging, U2OS cells were cultured in NEST glass dishes (10 mm in diameter). Before imaging, cells were treated with 1 µg/mL hoechst dye for 20 min, and then washed with DPBS for 3 times to remove the dye. Fresh culture medium was supplemented to cells afterwards. Cells were imaged using the Leica SP5 fluorescence confocal microscope or GE DeltaVision elite fluorescence microscope.

Images were processed and analyzed using ImageJ software or LAS AF Lite software. Multi-layer images were scanned using Zeiss LSM880 fluorescence confocal microscope and used for 3D reconstruction with Imaris software.

Fluorescence overlap coefficient of IRS1 condensates and downstream signaling proteins were analyzed using the Image-Pro Plus software (Media Cybernetics, Rockville, MD, USA).

### Fluorescence recovery after photobleaching

For photobleaching experiments, U2OS cells were seeded onto NEST glass dishes (10 mm in diameter) and transfected with indicated plasmids. A 488 nm laser at excitation intensity of Leica SP5 maximum power was used to photo-bleach regions of interest corresponding to individual condensates in the samples. The fluorescence intensity was monitored before and after photobleaching with time interval of few seconds.

### Statistical analysis

Data were analyzed using Prism software (GraphPad, San Diego, CA, USA). Comparisons of two groups and multiple groups were carried out via *t* test, one-way ANOVA, or two-way ANOVA, respectively. Statistical significance was considered for differences at *p* < 0.05.

## Supplementary information

Supplementary Information

Supplementary Video S1A

Supplementary Video S1B

Supplementary Video S1C

Supplementary Video S1D

## References

[1] Saltiel AR (2021). Insulin signaling in health and disease. J. Clin. Invest.

[2] Czech MP (2017). Insulin action and resistance in obesity and type 2 diabetes. Nat. Med..

[3] Haeusler RA, McGraw TE, Accili D (2018). Biochemical and cellular properties of insulin receptor signalling. Nat. Rev. Mol. Cell Biol..

[4] Hopkins BD, Goncalves MD, Cantley LC (2020). Insulin-PI3K signalling: an evolutionarily insulated metabolic driver of cancer. Nat. Rev. Endocrinol..

[5] Vanhaesebroeck B, Alessi DR (2000). The PI3K-PDK1 connection: more than just a road to PKB. Biochem J..

[6] Cohen P, Frame S (2001). The renaissance of GSK3. Nat. Rev. Mol. Cell Biol..

[7] Li Q (2019). The protein phosphatase 1 complex is a direct target of AKT that links insulin signaling to hepatic glycogen deposition. Cell Rep..

[8] Rena G, Guo S, Cichy SC, Unterman TG, Cohen P (1999). Phosphorylation of the transcription factor forkhead family member FKHR by protein kinase B. J. Biol. Chem..

[9] Titchenell PM, Lazar MA, Birnbaum MJ (2017). Unraveling the regulation of hepatic metabolism by insulin. Trends Endocrinol. Metab..

[10] Sano H (2003). Insulin-stimulated phosphorylation of a Rab GTPase-activating protein regulates GLUT4 translocation. J. Biol. Chem..

[11] Chen S, Wasserman DH, MacKintosh C, Sakamoto K (2011). Mice with AS160/TBC1D4-Thr649Ala knockin mutation are glucose intolerant with reduced insulin sensitivity and altered GLUT4 trafficking. Cell Metab..

[12] Chen Q (2019). Targeting RalGAPalpha1 in skeletal muscle to simultaneously improve postprandial glucose and lipid control. Sci. Adv..

[13] Quan C (2020). A PKB-SPEG signaling nexus links insulin resistance with diabetic cardiomyopathy by regulating calcium homeostasis. Nat. Commun..

[14] Shin Y, Brangwynne CP (2017). Liquid phase condensation in cell physiology and disease. Science.

[15] Zhang H (2020). Liquid-liquid phase separation in biology: mechanisms, physiological functions and human diseases. Sci. China Life Sci..

[16] Banani SF, Lee HO, Hyman AA, Rosen MK (2017). Biomolecular condensates: organizers of cellular biochemistry. Nat. Rev. Mol. Cell Biol..

[17] Zhang G, Wang Z, Du Z, Zhang H (2018). mTOR regulates phase separation of PGL granules to modulate their autophagic degradation. Cell.

[18] Xiao Q, McAtee CK, Su X (2022). Phase separation in immune signalling. Nat. Rev. Immunol..

[19] Schaefer KN, Peifer M (2019). Wnt/Beta-catenin signaling regulation and a role for biomolecular condensates. Dev. Cell.

[20] van Mierlo G (2021). Predicting protein condensate formation using machine learning. Cell Rep..

[21] Shin Y (2017). Spatiotemporal control of intracellular phase transitions using light-activated optoDroplets. Cell.

[22] Baroni MG (2001). The G972R variant of the insulin receptor substrate-1 (IRS-1) gene, body fat distribution and insulin-resistance. Diabetologia.

[23] Alberti S, Gladfelter A, Mittag T (2019). Considerations and challenges in studying liquid-liquid phase separation and biomolecular condensates. Cell.

[24] Fernandez LA (2009). YAP1 is amplified and up-regulated in hedgehog-associated medulloblastomas and mediates Sonic hedgehog-driven neural precursor proliferation. Genes Dev..

[25] Palomer X, Pizarro-Delgado J, Barroso E, Vazquez-Carrera M (2018). Palmitic and oleic acid: the yin and yang of fatty acids in type 2 diabetes mellitus. Trends Endocrinol. Metab..

[26] Liu M, He S, Cheng L, Qu J, Xia J (2020). Phase-separated multienzyme biosynthesis. Biomacromolecules.

[27] Poudyal RR, Pir Cakmak F, Keating CD, Bevilacqua PC (2018). Physical principles and extant biology reveal roles for RNA-containing membraneless compartments in origins of life chemistry. Biochemistry.

[28] Yu J (1998). Regulation of the p85/p110 phosphatidylinositol 3’-kinase: stabilization and inhibition of the p110alpha catalytic subunit by the p85 regulatory subunit. Mol. Cell Biol..

[29] Takeuchi H (1998). PTB domain of insulin receptor substrate-1 binds inositol compounds. Biochem J..

[30] Andjelkovic M (1997). Role of translocation in the activation and function of protein kinase B. J. Biol. Chem..

[31] Kelly KL, Ruderman NB (1993). Insulin-stimulated phosphatidylinositol 3-kinase. Association with a 185-kDa tyrosine-phosphorylated protein (IRS-1) and localization in a low density membrane vesicle. J. Biol. Chem..

[32] Clark SF, Martin S, Carozzi AJ, Hill MM, James DE (1998). Intracellular localization of phosphatidylinositide 3-kinase and insulin receptor substrate-1 in adipocytes: potential involvement of a membrane skeleton. J. Cell Biol..

[33] Porter HA, Perry A, Kingsley C, Tran NL, Keegan AD (2013). IRS1 is highly expressed in localized breast tumors and regulates the sensitivity of breast cancer cells to chemotherapy, while IRS2 is highly expressed in invasive breast tumors. Cancer Lett..

[34] Bommer GT (2010). IRS1 regulation by Wnt/beta-catenin signaling and varied contribution of IRS1 to the neoplastic phenotype. J. Biol. Chem..

[35] Morcavallo A, Stefanello M, Iozzo RV, Belfiore A, Morrione A (2014). Ligand-mediated endocytosis and trafficking of the insulin-like growth factor receptor I and insulin receptor modulate receptor function. Front. Endocrinol..

[36] Alessi DR (1997). Characterization of a 3-phosphoinositide-dependent protein kinase which phosphorylates and activates protein kinase Balpha. Curr. Biol..

[37] Sarbassov DD, Guertin DA, Ali SM, Sabatini DM (2005). Phosphorylation and regulation of Akt/PKB by the rictor-mTOR complex. Science.

[38] Currie RA (1999). Role of phosphatidylinositol 3,4,5-trisphosphate in regulating the activity and localization of 3-phosphoinositide-dependent protein kinase-1. Biochem J..

[39] Liu P (2015). PtdIns(3,4,5)P3-dependent activation of the mTORC2 kinase complex. Cancer Discov..

[40] Calleja V, Laguerre M, Parker PJ, Larijani B (2009). Role of a novel PH-kinase domain interface in PKB/Akt regulation: structural mechanism for allosteric inhibition. PLoS Biol..

[41] Laustsen PG (2002). Lipoatrophic diabetes in Irs1(-/-)/Irs3(-/-) double knockout mice. Genes Dev..

[42] Copps KD, White MF (2012). Regulation of insulin sensitivity by serine/threonine phosphorylation of insulin receptor substrate proteins IRS1 and IRS2. Diabetologia.

[43] Schlessinger J (2000). Cell signaling by receptor tyrosine kinases. Cell.

