# Supplementary material for: Spatiotemporal regulation of insulin signaling by liquid–liquid phase separation
Source: Cell Discov. 2022 Jul 5;8:64. doi: 10.1038/s41421-022-00430-1 (PMC9256590; doi:10.1038/s41421-022-00430-1)

Spatiotemporal regulation of insulin signaling by liquid-liquid phase separation

Kun Zhou[1,2,3], Qiaoli Chen[1,2,3], Jiamou Chen[1], Derong Liang[1], Weikuan Feng[1], Minjun Liu[1], Qi Wang[1], Ruizhen Wang[1], Qian Ouyang[1], Chao Quan[1,2]* and Shuai Chen[1,2]*

SUPPLEMENTARY INFORMATION

Supplementary Table S1 LLPS prediction of insulin-related proteins using PSAP

KEGG pathway analysis revealed 173 insulin-related proteins including the receptor, mediators and effectors of insulin signaling pathway, which were subjected to analyses using the predictor PSAP to obtain their LLPS scores.

Supplementary Table S1

UniprotKB_ID	Uniprot ID	PASP score
IRS4_HUMAN	O14654	0.01750
RXRA_HUMAN	P19793	0.01583
CIP4_HUMAN	Q15642	0.01333
SHC1_HUMAN	P29353	0.01333
SHC4_HUMAN	Q6S5L8	0.01083
KAP1_HUMAN	P31321	0.00917
CRKL_HUMAN	P46109	0.00917
PI51A_HUMAN	Q99755	0.00917
AAKG2_HUMAN	Q9UGJ0	0.00833
ATF2_HUMAN	P15336	0.00750
CBLB_HUMAN	Q13191	0.00667
RPGF1_HUMAN	Q13905	0.00667
CREB5_HUMAN	Q02930	0.00667
ATX1_HUMAN	P54253	0.00667
TIF1A_HUMAN	O15164	0.00667
KAP3_HUMAN	P31323	0.00583
CRK_HUMAN	P46108	0.00583
PALLD_HUMAN	Q8WX93	0.00583
SOS2_HUMAN	Q07890	0.00500
FOXO1_HUMAN	Q12778	0.00500
P85B_HUMAN	O00459	0.00500
MYT1_HUMAN	Q01538	0.00500
PRGC1_HUMAN	Q9UBK2	0.00417
WEE1_HUMAN	P30291	0.00417
BRAF_HUMAN	P15056	0.00333
MKNK1_HUMAN	Q9BUB5	0.00333
PKN1_HUMAN	Q16512	0.00333
MDM2_HUMAN	Q00987	0.00333
KS6B1_HUMAN	P23443	0.00250
CBL_HUMAN	P22681	0.00250
GSK3B_HUMAN	P49841	0.00250
ARAF_HUMAN	P10398	0.00250
SOCS6_HUMAN	O14544	0.00250
CREB1_HUMAN	P16220	0.00250
IRS1_HUMAN	P35568	0.00167
PPR3B_HUMAN	Q86XI6	0.00167
SRBS1_HUMAN	Q9BX66	0.00167
ELK1_HUMAN	P19419	0.00167
KAP2_HUMAN	P13861	0.00167
MKNK2_HUMAN	Q9HBH9	0.00167
AAKB1_HUMAN	Q9Y478	0.00167
CALL3_HUMAN	P27482	0.00167
CASP9_HUMAN	P55211	0.00167
ATF6B_HUMAN	Q99941	0.00167
NEMO_HUMAN	Q9Y6K9	0.00167
TBCD4_HUMAN	O60343	0.00167
BAX_HUMAN	Q07812	0.00167
IRS2_HUMAN	Q9Y4H2	0.00083
KPCA_HUMAN	P17252	0.00083
KPCI_HUMAN	P41743	0.00083
RAF1_HUMAN	P04049	0.00083
KS6B2_HUMAN	Q9UBS0	0.00083
PPR3A_HUMAN	Q16821	0.00083

PDE3B_HUMAN	Q13370	0.00083
MK03_HUMAN	P27361	0.00083
AKT3_HUMAN	Q9Y243	0.00083
CBLC_HUMAN	Q9ULV8	0.00083
LIPS_HUMAN	Q05469	0.00083
SOCS1_HUMAN	O15524	0.00083
MP2K1_HUMAN	Q02750	0.00083
PTN1_HUMAN	P18031	0.00083
AKT2_HUMAN	P31751	0.00083
EXOC7_HUMAN	Q9UPT5	0.00083
KPB1_HUMAN	P46020	0.00083
IF4E2_HUMAN	O60573	0.00083
RS6_HUMAN	P62753	0.00083
IF4E_HUMAN	P06730	0.00083
FLOT1_HUMAN	O75955	0.00083
RASN_HUMAN	P01111	0.00083
PHKG2_HUMAN	P15735	0.00083
PKN3_HUMAN	Q6P5Z2	0.00083
GRDN_HUMAN	Q3V6T2	0.00083
CCNA2_HUMAN	P20248	0.00083
GSK3A_HUMAN	P49840	0.00083
AKTS1_HUMAN	Q96B36	0.00083
M3K8_HUMAN	P41279	0.00083
XIAP_HUMAN	P98170	0.00083
TSC1_HUMAN	Q92574	0.00000
SRBP1_HUMAN	P36956	0.00000
PDPK1_HUMAN	O15530	0.00000
SHIP1_HUMAN	Q92835	0.00000
FLOT2_HUMAN	Q14254	0.00000
BAD_HUMAN	Q92934	0.00000
PI3R5_HUMAN	Q8WYR1	0.00000
PPR3C_HUMAN	Q9UQK1	0.00000
PDE3A_HUMAN	Q14432	0.00000
TSC2_HUMAN	P49815	0.00000
PTPRF_HUMAN	P10586	0.00000
SHC3_HUMAN	Q92529	0.00000
SHC2_HUMAN	P98077	0.00000
FAS_HUMAN	P49327	0.00000
HXK2_HUMAN	P52789	0.00000
HXK3_HUMAN	P52790	0.00000
ACACB_HUMAN	O00763	0.00000
M4K2_HUMAN	Q12851	0.00000
MP2K2_HUMAN	P36507	0.00000
SH2B2_HUMAN	O14492	0.00000
RPTOR_HUMAN	Q8N122	0.00000
GYS2_HUMAN	P54840	0.00000
PCKGM_HUMAN	Q16822	0.00000
ACACA_HUMAN	Q13085	0.00000
KPB2_HUMAN	P46019	0.00000
PYGL_HUMAN	P06737	0.00000
INSR_HUMAN	P06213	0.00000
P85A_HUMAN	P27986	0.00000
SOCS3_HUMAN	O14543	0.00000
PYGM_HUMAN	P11217	0.00000
PK3CD_HUMAN	O00329	0.00000
HXK1_HUMAN	P19367	0.00000

HXK4_HUMAN	P35557	0.00000
PRKX_HUMAN	P51817	0.00000
AAKG3_HUMAN	Q9UGI9	0.00000
AAKG1_HUMAN	P54619	0.00000
PPR3D_HUMAN	O95685	0.00000
KPCZ_HUMAN	Q05513	0.00000
GYS1_HUMAN	P13807	0.00000
PK3CA_HUMAN	P42336	0.00000
AAPK1_HUMAN	Q13131	0.00000
PCX4_HUMAN	Q63HM2	0.00000
RHOQ_HUMAN	P17081	0.00000
S19A1_HUMAN	P41440	0.00000
KPYR_HUMAN	P30613	0.00000
MK10_HUMAN	P53779	0.00000
PYGB_HUMAN	P11216	0.00000
PP1G_HUMAN	P36873	0.00000
KPBB_HUMAN	Q93100	0.00000
MK01_HUMAN	P28482	0.00000
AKT1_HUMAN	P31749	0.00000
PK3CG_HUMAN	P48736	0.00000
MK09_HUMAN	P45984	0.00000
PCKGC_HUMAN	P35558	0.00000
AAPK2_HUMAN	P54646	0.00000
MK08_HUMAN	P45983	0.00000
PP1A_HUMAN	P62136	0.00000
PP1B_HUMAN	P62140	0.00000
MTOR_HUMAN	P42345	0.00000
F16P1_HUMAN	P09467	0.00000
PHKG1_HUMAN	Q16816	0.00000
KAPCG_HUMAN	P22612	0.00000
F16P2_HUMAN	O00757	0.00000
INP5K_HUMAN	Q9BT40	0.00000
PK3CB_HUMAN	P42338	0.00000
RASK_HUMAN	P01116	0.00000
SOCS2_HUMAN	O14508	0.00000
IKKB_HUMAN	O14920	0.00000
AAKB2_HUMAN	O43741	0.00000
P55G_HUMAN	Q92569	0.00000
RASH_HUMAN	P01112	0.00000
G6PC2_HUMAN	Q9NQR9	0.00000
G6PC_HUMAN	P35575	0.00000
SOCS4_HUMAN	Q8WXH5	0.00000
I4E1B_HUMAN	A6NMX2	0.00000
KAPCA_HUMAN	P17612	0.00000
KAPCB_HUMAN	P22694	0.00000
RHEB_HUMAN	Q15382	0.00000
CALL5_HUMAN	Q9NZT1	0.00000
CALL6_HUMAN	Q8TD86	0.00000
PKN2_HUMAN	Q16513	0.00000
SGK1_HUMAN	O00141	0.00000
SGK2_HUMAN	Q9HBY8	0.00000
SGK3_HUMAN	Q96BR1	0.00000
CREB3_HUMAN	O43889	0.00000
ATF4_HUMAN	P18848	0.00000
NR4A1_HUMAN	P22736	0.00000
ACLY_HUMAN	p53396	0.00000

CDK2_HUMAN	P24941	0.00000
NOS3_HUMAN	P29474	0.00000
IKKA_HUMAN	O15111	0.00000
CDN1A_HUMAN	P38936	0.00000
CDN1B_HUMAN	P46527	0.00000
NCF1_HUMAN	P14598	0.00000
SCF_HUMAN	P21583	0.00000
SKP2_HUMAN	Q13309	0.00000

SUPPLEMENTARY FIGURE LEGENDS

Supplementary Figure S1 FRAP analyses of insulin-related proteins with LLPS potential

a-b. FRAP analysis of optoDroplets of insulin-related proteins in Cos-7 cells. The optoDroplets subjected to FRAP analysis were highlighted in insets and indicated with red arrows. Scale bars indicate 10 μm in length.

c. Quantification of FRAP analyses of optoDroplets of insulin-related proteins in Cos-7 cells. n = 3-7. Representative images were shown in Figure 1e and Supplementary Fig. S1a-b.

Supplementary Figure S2 *In vitro* fusion of droplets of insulin-related proteins with LLPS potential

a. Fusion of droplets of His-tagged BAD-GFP, GSK3β-GFP, P27-GFP and PRAS40-GFP. Red arrows showed the two droplets that fused during the experimental period. Time Zero started at 90 sec after the addition of PEG8000. Scale bars indicate 10 μm in length.

b. *In vitro* LLPS assay with 20 μM recombinant FITC-labelled His-tagged proteins, GSK3β, PRAS40, BAD, P27 with 3% PEG8000. Scale bars indicate 10 μm in length.

c. *In vitro* LLPS assay with different concentrations of recombinant FITC-labelled Flag-IRS1 with 3% PEG8000. Scale bars indicate 10 μm in length.

Supplementary Figure S3 Subcellular distribution of IRS1-GFP condensates in cells

a. Subcellular localization of key insulin signaling components. mCherry-IR, mCherry-p85, mCherry-PKBβ and IRS1-GFP were expressed in Cos-7 cells. Scale bars indicate 10 μm in length.

b. 3D reconstruction of IRS1-GFP condensates in U2OS cells. Two different image angles were shown. The droplets in the orange dotted circle were oblique clipped and shown in the lower right corner.

c. 3D reconstruction of IRS1-GFP condensates in mouse primary hepatocytes. Three different image angles were shown.

d. Distribution of endogenous IRS1 in the cytosol and nucleus of L6 muscle cells.

e. Distribution of endogenous IRS1 in the cytosol and nucleus of U2OS cells.

f. Distribution of exogenous IRS1-GFP in the cytosol and nucleus of U2OS cells.

Supplementary Figure S4 Subcellular localization of IRS1-GFP condensates in cells

a-b. Subcellular localization of IRS1-GFP condensates in U2OS cells. Cells transfected with IRS1-GFP were stained with 1,1'-dioctadecyl-3,3,3',3'-tetramethylindocarbocyanine perchlorate (DiI, Beyotime Biotechnology) for plasma membrane. In the figure A, images were taken using the Leica SP5 confocal microscope. Scale bars indicate 10 μm in length. In the figure B, images were taken from the same cell first under the epifluorescence (Epi) mode and then under the TIRF mode using the GE DeltaVision OMX microscope. Scale bars indicate 10 μm in length.

c. Co-localization of IRS1-GFP condensates with organelles in U2OS cells. Cells transfected with IRS1-GFP were stained with markers for mitochondria (TOM20), endoplasmic reticulum (PDI), Golgi (RCAS1), early endosome (Rab5), late endosome (Rab7), lysosome (LAMP1) and F-actin. Scale bars indicate 10 μm in length.

Supplementary Figure S5 Dynamics of endogenous IRS1 condensates in cells

a-b. Formation of endogenous IRS1 condensates in primary brown adipocytes upon insulin stimulation. a, representative images. b, fitted curves of cell distribution in terms of IRS1 condensate (puncta) number per cell. The values were fitted into curves using cubic spline. n = 21-45. Scale bars indicate 10 μm in length.

c-d. Formation of endogenous IRS1 condensates in Cos-7 cells upon insulin stimulation. c, representative images. d, fitted curves of cell distribution in terms of IRS1 condensate (puncta) number per cell. The values were fitted into curves using polynomials. n = 22. Scale bars indicate 10 μm in length.

e. L6 muscle cells were treated with 0, 200 and 400 μM of palmitate for 24 hrs before stimulated with or without insulin. Ser473 phosphorylation of PKB was measured via immunoblotting.

f-g. Effects of palmitate treatment on insulin-induced formation of IRS1 condensates in primary brown adipocytes. Cells were treated with or without palmitate for 24 hrs before stimulated with or without insulin. f, representative images. Scale bars indicate 10 μm in length. g, quantification of IRS1 puncta per cell. n = 50. The data are given as the mean ± SEM. Statistical analyses were carried out via two-way ANOVA. *** indicates $p < 0.001$. n.s., not significant.

h-j. Effects of the PKC inhibitor Gö6983 on PKB phosphorylation and IRS1 puncta formation in U2OS cells. Cells were treated with or without palmitate for 24 hrs before stimulated with or without insulin in the presence or absence of Gö6983. h, representative images of IRS1 puncta. Scale bars indicate 10 μm in length. i, quantification of IRS1 puncta per cell. j, immunoblotting analysis of PKB phosphorylation. n = 55-61. The data are given as the mean ± SEM. Statistical analyses were carried out via two-way ANOVA. *** indicates $p < 0.001$.

Supplementary Figure S6 Recruitment of signaling molecules into IRS1 condensates

The IRS1-GFP was co-expressed with mCherry-p85, mCherry-p110, PIP2 sensor, mCherry-PDK1, mCherry-SIN1, PIP3 sensor and mCherry-PKBβ in cells. Overlap coefficient between IRS1-GFP and these signaling molecules was determined within the IRS1 condensates. Representative images were shown in Figure 5b-h. Quantification of overlap coefficient of IRS1-GFP/signaling molecules in the IRS1 condensates was shown here. n = 44-73 (IRS1/p85), 63-67 (IRS1/p110), 68-71 (IRS1/PIP2 sensor), 84 (IRS1/PDK1), 81-155 (IRS1/SIN1), 86 (IRS1/PIP3 sensor), 123-196 (IRS1/PKBβ). The data are given as the mean ± SEM. Statistical analyses were carried out via t-test. *** indicates $p < 0.001$. n.s., not significant.

Supplementary Figure S7 Localization of PIP3 sensor in cells in response to insulin

Cos-7 cells transfected with PIP3 sensor was stimulated with or without insulin. Localization of PIP3 sensor was detected using a confocal microscope. Scale bars indicate 10 μm in length.

Supplementary Figure S8 Recruitment of mCherry-p85 into WT and mutant IRS1 condensates

The WT and mutant IRS1-GFP were co-expressed with mCherry-p85 in U2OS cells. Overlap coefficient between IRS1-GFP and mCherry-p85 was determined within the IRS1 condensates. a, representative images. b, quantification of IRS1-GFP/mCherry-p85 overlap coefficient in the IRS1 condensates. n = 99-111. The data are given as the mean ± SEM. Statistical analyses were carried out via one-way ANOVA. *** indicates $p < 0.001$. n.s., not significant.

SUPPLEMENTARY VIDEO LEGENDS

Supplementary Video S1 Induction of optoDroplets of insulin-related proteins with blue light

a-d. Formation of optoDroplets for optoP47PHOX (a), optoACLY (b), optoIKKα (c) and optoCyclinA (d) upon induction with blue light in Cos-7 cells.

Supplementary Fig. S1

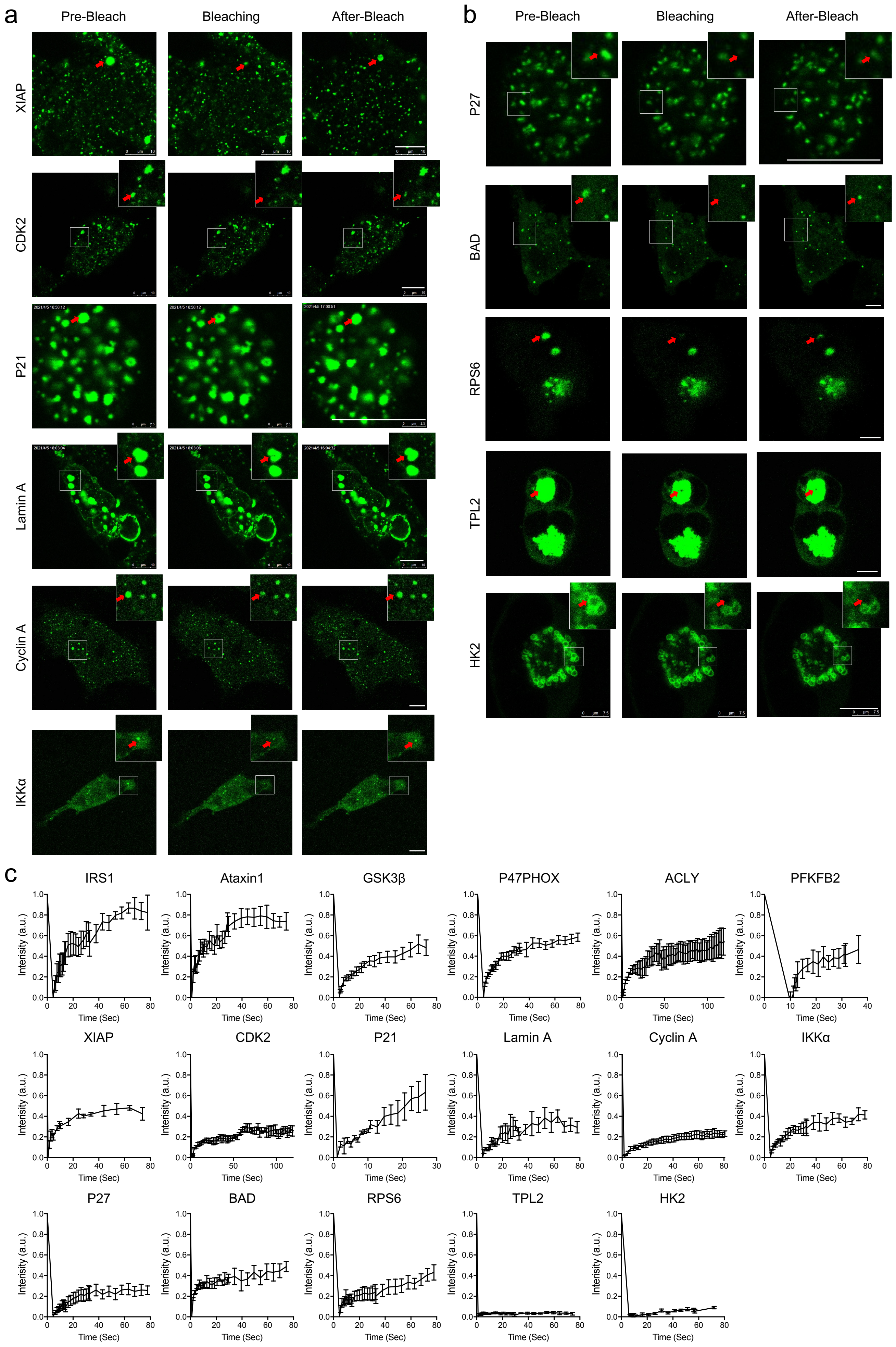

Supplementary Fig. S2

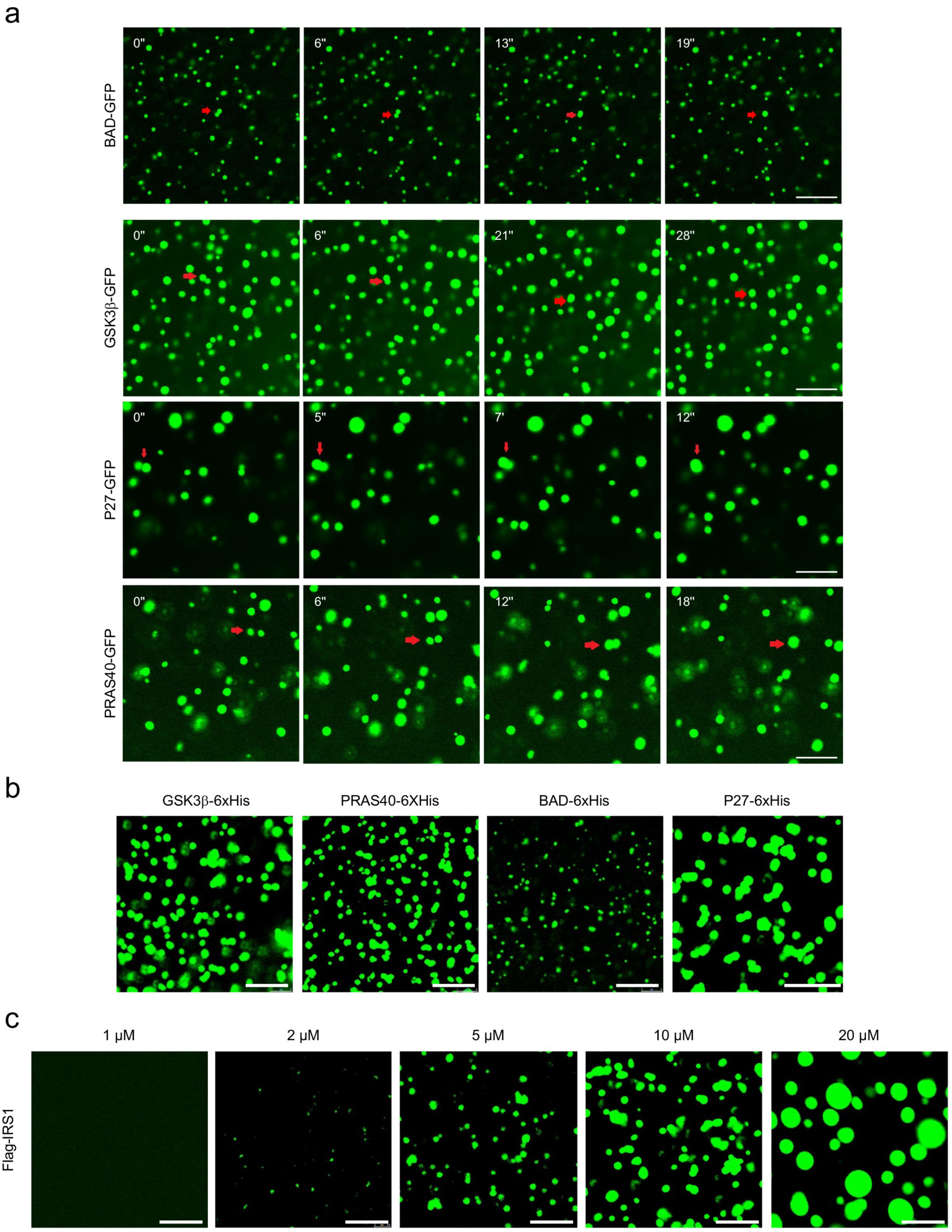

Supplementary Fig. S3

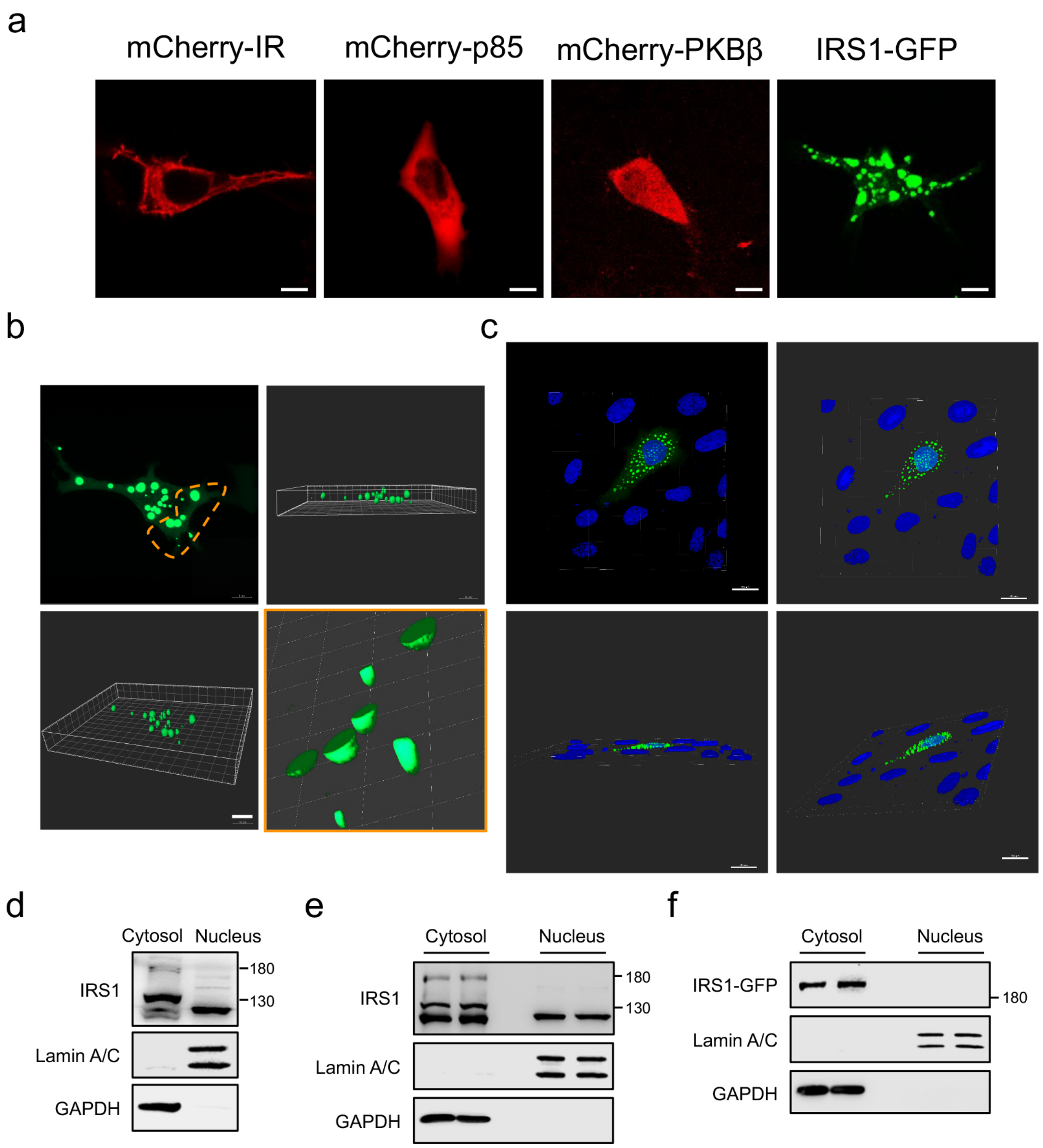

Supplementary Fig. S4

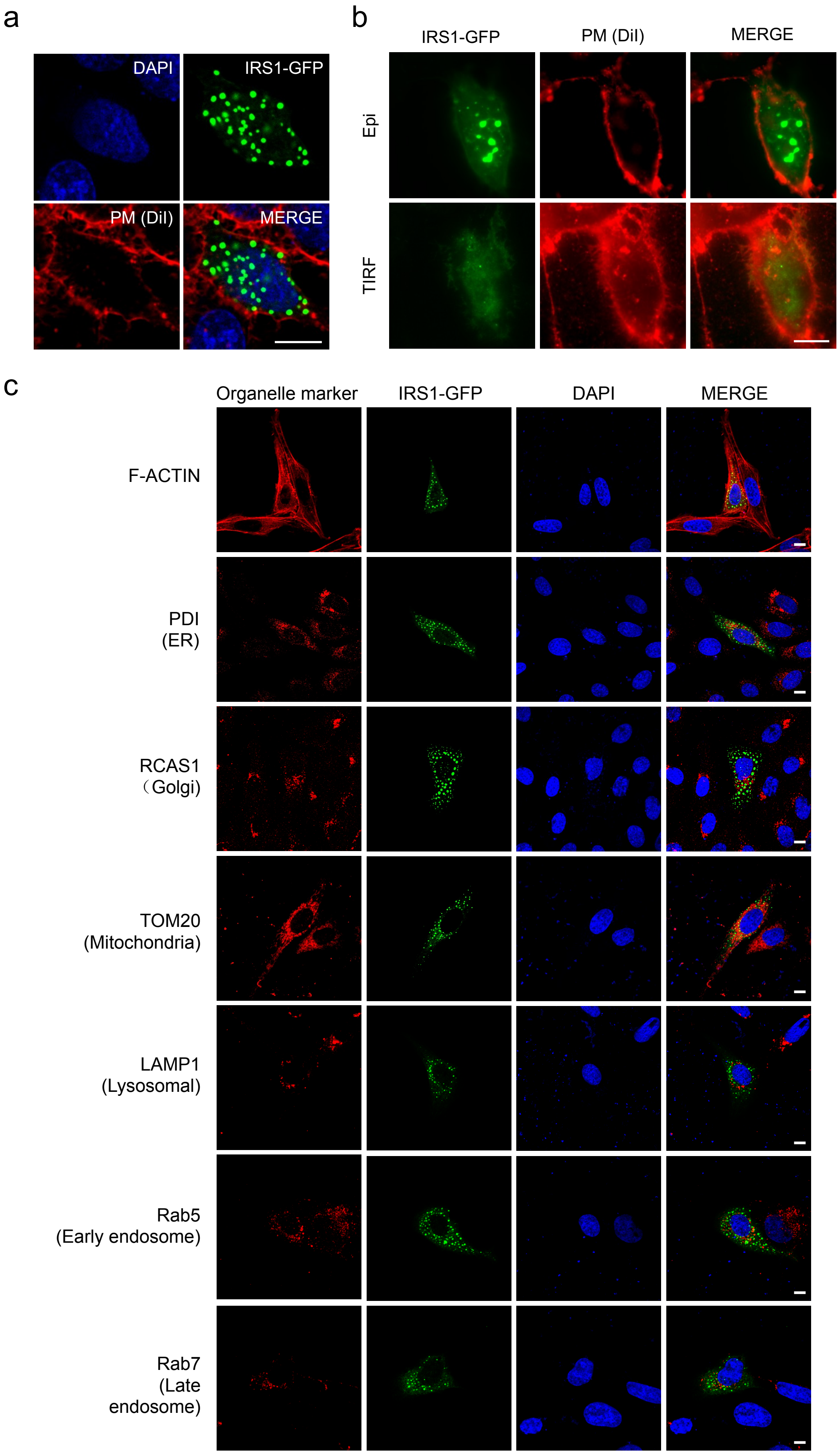

Supplementary Fig. S5

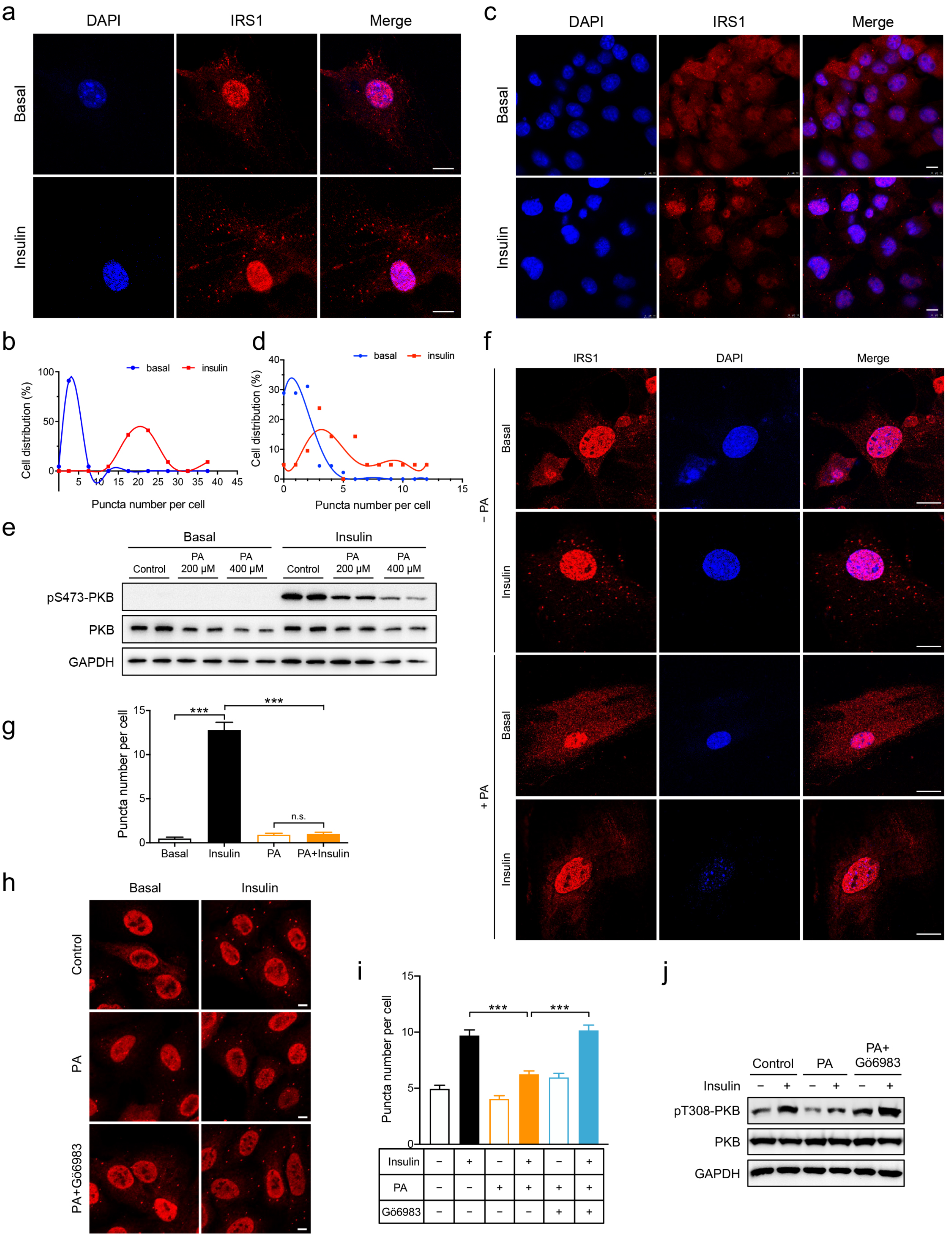

Supplementary Fig. S6

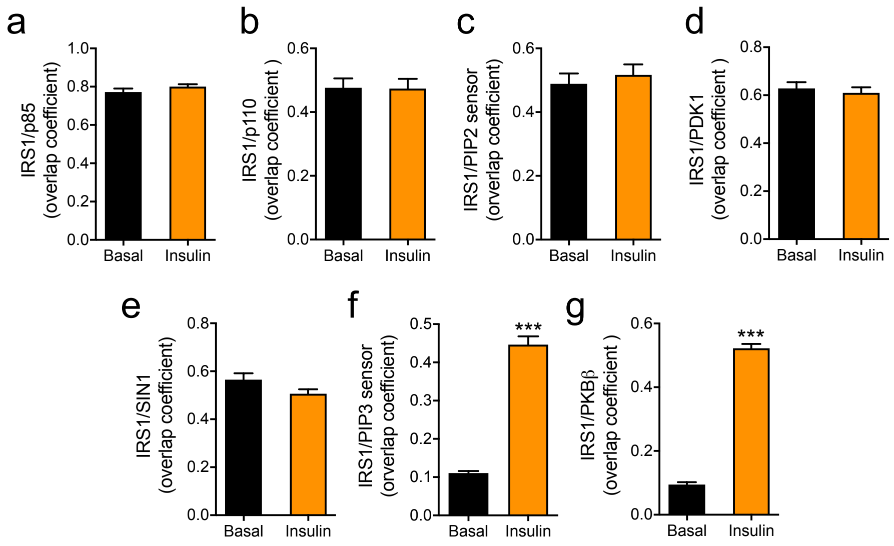

Supplementary Fig. S7

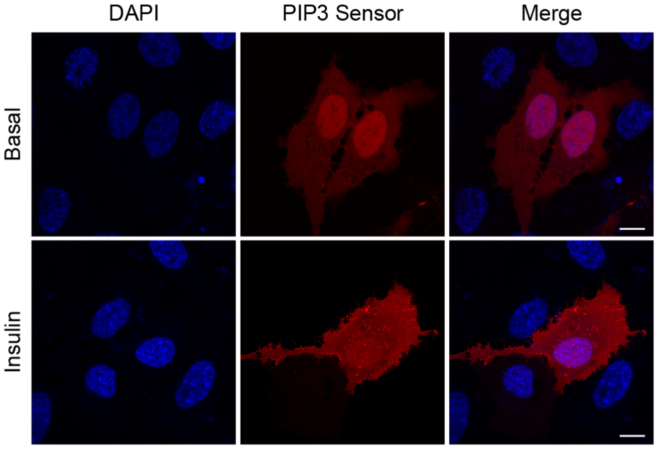

Supplementary Fig. S8

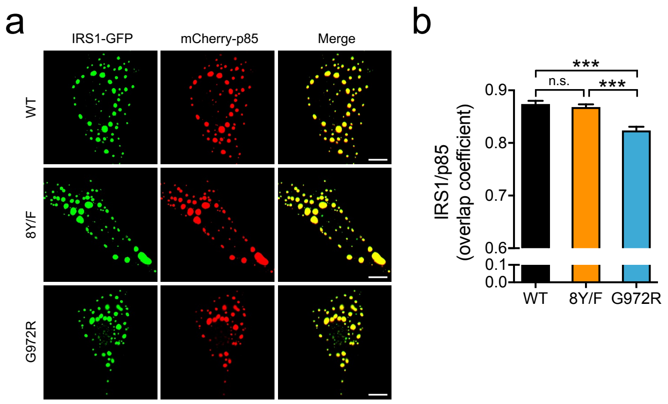

Supplement: Supplementary file 1 — Supplementary Information [file 41421_2022_430_MOESM1_ESM.pdf]
